# Undoing of firing rate adaptation enables invariant population codes

**DOI:** 10.1126/sciadv.aeg3535

**Published:** 2026-08-19

**Authors:** Sofia C. Brandão, Luisa Ramirez, Pascal Züfle, Alexander M. Walter, Marion Silies, Carlotta Martelli

**Affiliations:** ^1^ Institute of Developmental Biology and Neurobiology, Johannes Gutenberg University, Mainz, Germany.; ^2^ Institute for Quantitative and Computational Biosciences (IQCB), Johannes Gutenberg University, Mainz, Germany.; ^3^ Department of Neuroscience, Faculty of Health and Medical Sciences, University of Copenhagen, Copenhagen, Denmark.

## Abstract

Neural adaptation supports coding efficiency by tuning responses to prevailing stimulus statistics. However, when information is represented by populations of neurons, adaptation of individual units could degrade behaviorally relevant signals. Here, we investigate how the fly olfactory system implements adaptation to background odors in olfactory receptor neurons (ORNs) and the consequences for combinatorial coding in downstream circuits. We show that adaptation of ORN firing rate is compensated at the axon terminal, where inhibitory presynaptic feedback supports background-invariant calcium responses to increases (but not decreases) in odor concentration. Computational analyses demonstrate that such invariance requires an adaptation strategy in ORNs that shifts response amplitude rather than sensitivity, diverging from efficient coding principles in single neurons. Downstream, postsynaptic projection neurons also exhibit background-invariant responses through presynaptic plasticity involving Unc13 proteins. Thus, the olfactory circuit restores stable population codes by undoing peripheral firing rate adaptation, enabling an asymmetric contrast representation that preserves stimulus identity across backgrounds.

## INTRODUCTION

Neural adaptation is an essential strategy of sensory systems that allows the encoding of stimuli over a range of intensities that exceeds the limits of an individual sensory cell (*1*). To do so, neurons tune their stimulus-response function to the relevant statistics of the stimulus (*2*–*4*). For instance, adaptation to increasing background luminance leads to a shift in the response curve of fly photoreceptors toward higher luminance (*5*). Such response strategies are not restricted to receptors but generalize to higher-order neurons within circuits that support efficient coding through normative computations (*6*, *7*). The simplest formulation of the efficient coding theory for a single receptor neuron predicts that a shift in neuron sensitivity that matches the mean stimulus optimizes the encoding of relevant information within the neuron’s dynamic range (*8*). Such shifts in sensitivity have been observed in several sensory systems, from audition to vision, in artificial and natural conditions (*3*, *5*, *6*, *9*). Whether shifting sensory neuron sensitivity is a key adaptive strategy also for odor coding remains unclear. The olfactory system is by design endowed with a large array of sensory receptors with different affinities to the same odorant (*10*, *11*). When the stimulus intensity (concentration) increases, the more sensitive receptors saturate, while new ones with lower affinity are recruited. Therefore, the system as a whole is rarely out of its broad dynamic range, raising the question of whether shifting receptor sensitivity is the main adaptive strategy of an olfactory system. While the benefits of adaptation in single neurons have been widely discussed and generalized across modalities (*12*, *13*), adaptation strategies at the population level might optimize different functions. For example, neuronal adaptation in the visual cortex reduces correlations, improving coding accuracy at the population level (*14*). Similarly, neural representations of odors in second-order neurons change over time, decorrelating population responses, providing a mechanism to disentangle odor mixtures (*15*). In general, adaptive strategies that optimize coding in single neurons might not necessarily optimize information coding at the population level and vice versa.

In both vertebrates and invertebrates, the firing rate of olfactory receptor neurons (ORNs) decreases upon persistent stimulation (*16*–*22*), inducing a change in their response curves. The coding function of this adaptive change remains unclear. Compared to background adaptation in photoreceptors, ORNs in both insects and vertebrates do not seem to markedly shift their sensitivity after adaptation to a background [reviewed in (*18*, *23*)]. Rather, background stimuli decrease the ORN dynamic range, reducing their maximum firing rate. Under dynamic stimulus conditions, the fly ORNs adapt their firing rate to the stimulus mean and show contrast sensitivity within a moderate range of stimulus intensities (*24*, *25*). However, contrast computation in single ORNs before the encoding of odor identity across the ORN population could interfere with information transmission to downstream circuits.

Combinatorial coding is a key property of the olfactory system (*10*, *26*). Most odorants activate combinations of ORNs on the basis of the sensitivity of the odorant receptor that they express. ORNs expressing the same receptor send axons to the same olfactory glomerulus, where they connect to projection neurons (PNs) in insects and mitral/tuft cells in vertebrates (*27*). The combinatorial representation of an odor across ORNs is not simply inherited by these second-order olfactory neurons. Synaptic depression in these first-order synapses (*28*, *29*) and lateral inhibition (*30*, *31*) mediated by local neurons (LNs) modulate odor representations in the output of the insect antennal lobe (AL) (*32*–*35*) and the vertebrate olfactory bulb (*36*). The role of these two circuit mechanisms for odor coding in background-adapted conditions remains unclear. Calcium responses measured in ORN axons do not show the adaptive changes observed in firing rates (*37*), raising the question of whether circuit computations within and across glomeruli might affect how adapted firing rates drive synaptic output and, therefore, the odor information sent to downstream circuits in the presence of a background.

Here, we combine computational and experimental approaches in *Drosophila* to understand how ORN adaptive properties shape odor representations in populations of second-order olfactory neurons, the PNs. We show that ORN firing rate adaptation is compensated at the axon terminal, leading to invariant calcium dynamics, and that this compensation predominantly depends on GABAergic feedback inhibition. We show analytically and with simulations that background invariance can be achieved for any background only if the firing rate of individual ORNs adapts by a shift in amplitude rather than in sensitivity. This finding assigns a functional role to ORN firing rate adaptation that diverges from efficient stimulus encoding in single neurons and supports contrast encoding at the population level. We further show that background invariance is specific for ON stimuli (increase in concentrations compared to the background), creating an asymmetry between ON and OFF odor representations. Such asymmetric invariance persists in postsynaptic PNs and is disrupted by mutation of a modulatory domain of Unc13, suggesting a role of presynaptic plasticity in propagating robust odor information. We propose that the undoing of ORN firing rate adaptation allows a background-invariant representation of odor identity for ON stimuli, enhancing the separation of positive and negative contrast in PN populations.

## RESULTS

### ORNs transform adapted firing rate responses to odor stimuli into background-invariant calcium representations

**Fig. 1.**
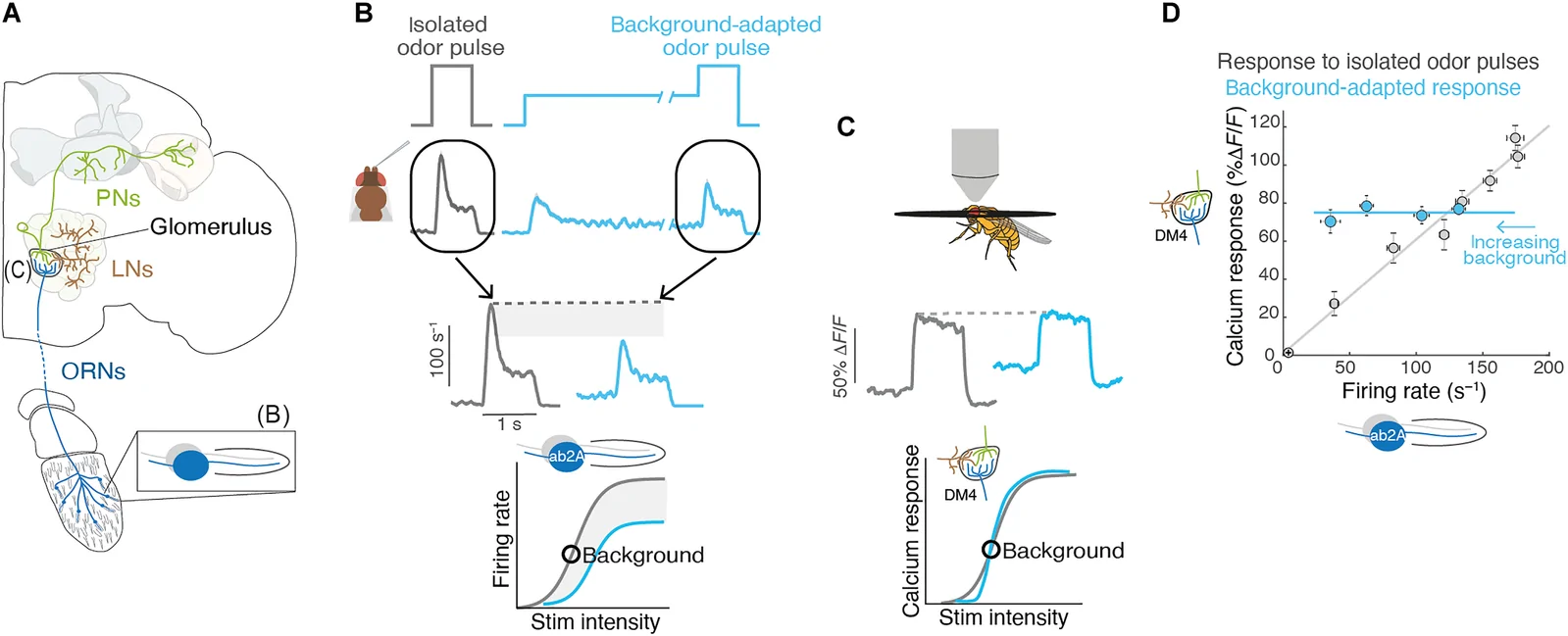
Undoing of firing rate adaptation at central synapses. (**A**) Schematics of the olfactory pathway. ORNs located in the antenna extend their axons to a specific glomerulus in the AL and make synapses onto LNs and uniglomerular PNs. PNs’ axons innervate the LH and the calyx of the MB. The labels (b and c) indicate the location of recordings of the corresponding panels. (**B**) Top: Schematics of the stimulus protocol and firing rate response of the ab2A ORNs in control and background-adapted conditions [data adapted from (*37*)]. Bottom: Schematics of the ORN firing response curve in control and background-adapted conditions. The black circle indicates the intensity of the background stimulus. (**C**) Top: ORN calcium responses induced by an odor puff in control and adapted conditions measured from the ab2A ORN axon terminals in the corresponding glomerulus DM4 [data adapted from (*37*)]. Bottom: Schematics of the ORN calcium response curve showing background-invariant responses. (**D**) Mean firing rate and mean calcium responses for odor puffs of different intensities tested in adapted (blue; same absolute ON amplitude presented in different backgrounds) and nonadapted (gray; multiple ON amplitudes) conditions [data from (*37*)]. The lines are linear regressions. Error bars indicate SE.

In flies, ORN dendrites innervate sensory organs called sensilla on the antennae and maxillary palps (Fig. 1A). ORNs expressing the same receptor target an individual glomerulus of the AL (Fig. 1A). Response dynamics of ORN firing rates have been previously characterized by extracellular recordings from the dendritic site (*17*, *18*, *37*, *38*). An odor onset elicits a phasic firing rate response, which relaxes to an adapted steady state (Fig. 1B). As expected, in the adapted state, the neuron’s response to subsequent odor stimuli is weaker [Fig. 1B; data from (*37*)]. This firing activity drives calcium transients in the axon terminals of ORNs within the corresponding glomerulus, which can be quantified by two-photon imaging (Fig. 1C). We have previously shown that these presynaptic calcium signals exhibit sustained, rather than phasic, dynamics, and that their amplitude remains invariant to background odor levels despite adaptation of the firing rate [Fig. 1C; data from (*37*)]. This is true for both private odors that activate a single glomerulus and for public odors that activate multiple glomeruli (*37*). A new analysis of these previously published data reveals that, for isolated odor stimuli, presynaptic calcium transients depend linearly on firing rate, but this relationship flattens down under background-adapted conditions, leading to a background-invariant response [Fig. 1D; data from (*37*)]. These findings led us to hypothesize that presynaptic calcium dynamics and, therefore, synaptic release are regulated in an activity- and background-dependent manner.

### ORN presynaptic background invariance is asymmetric for ON and OFF stimuli

**Fig. 2.**
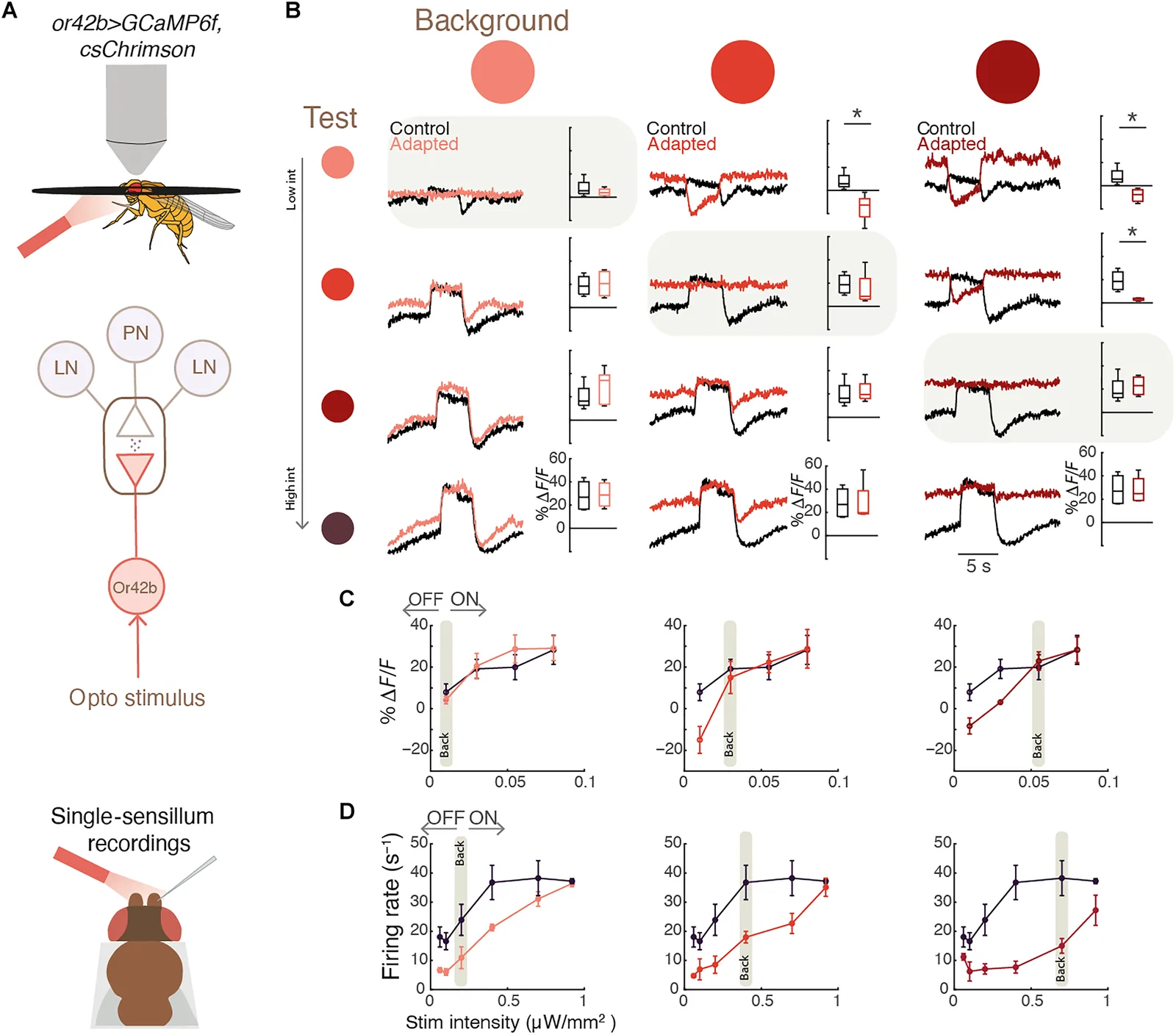
Asymmetric background invariance in ORNs upon optogenetic activation. (**A**) Schematics of the experimental protocol. (**B**) Mean calcium responses from ORN axon terminals in the glomerulus DM1 in control (black; no background) and adapted (shades of red) conditions. Columns represent different background intensities, and rows represent different intensities of the test stimulus (pulses). Test intensities are 0.01, 0.03, 0.055, and 0.08 μW/mm^2^ (from light to dark red). The plots on the diagonal (highlighted in gray) correspond to the same intensity of the test and background stimuli. Quadrants above this diagonal are OFF stimuli, and quadrants below are ON stimuli. Box plots show the median, quartiles, and min/max values of the response averaged in 3 s after test stimulus onset (*n* = 4 or 5, *P < 0.05). (**C**) Mean response plotted as a function of the test stimulus intensity. Error bars indicate SE. (**D**) Same as (C) but for firing rate responses (*n* = 3). A similar range of stimuli was used for the electrophysiology (0.06, 0.1, 0.2, 0.4, 0.7, and 0.92 μW/mm^2^). Gray boxes indicate the intensity of the background stimulus.

The background invariance observed in ORN axonal calcium suggests that these signals may encode absolute odor concentration. If this were the case, responses to both increases (ON) and decreases (OFF) in odor concentration should be background-invariant. To test this hypothesis, we used an optogenetic approach to mimic ORN odor responses while restricting background and test stimulations to a single ORN type (expressing the odorant receptor Or42b) that targets the glomerulus DM1 (Fig. 2A). We used several combinations of light intensities to test a range of background and test stimuli. ON stimuli elicited calcium transients of the same amplitude irrespective of background (Fig. 2, B and C). Notably, OFF stimuli always led to drops in calcium below the nonadapted response (Fig. 2, B and C). We conclude that ORN calcium responses encode the absolute concentration only for ON stimuli, a property that we name “asymmetric background invariance.” We further measured firing rate responses to optogenetic activation in the ab1 sensillum, which contains the Or42b-ORNs, to verify that these responses are, as expected, adapted to the stimulus background (Fig. 2D). Optogenetic activation leads to lower firing rates and a smaller dynamic range compared to odor stimuli (response saturates at around 40 Hz rather than the usual 250 Hz). Yet, as with odors, background adaptation leads to notable changes of the firing rate responses for both ON and OFF stimuli (Fig. 2D).

Together, optogenetic activation of a single ORN type recapitulates previous findings with private and public odorants (*37*), demonstrating that ORN calcium invariance is specific to ON stimuli and does not require inputs from multiple glomeruli. The differences between firing rate and calcium response properties suggest the existence of a presynaptic mechanism that compensates firing rate adaptation.

### Homeostatic feedback regulates ORN presynaptic calcium responses

**Fig. 3.**
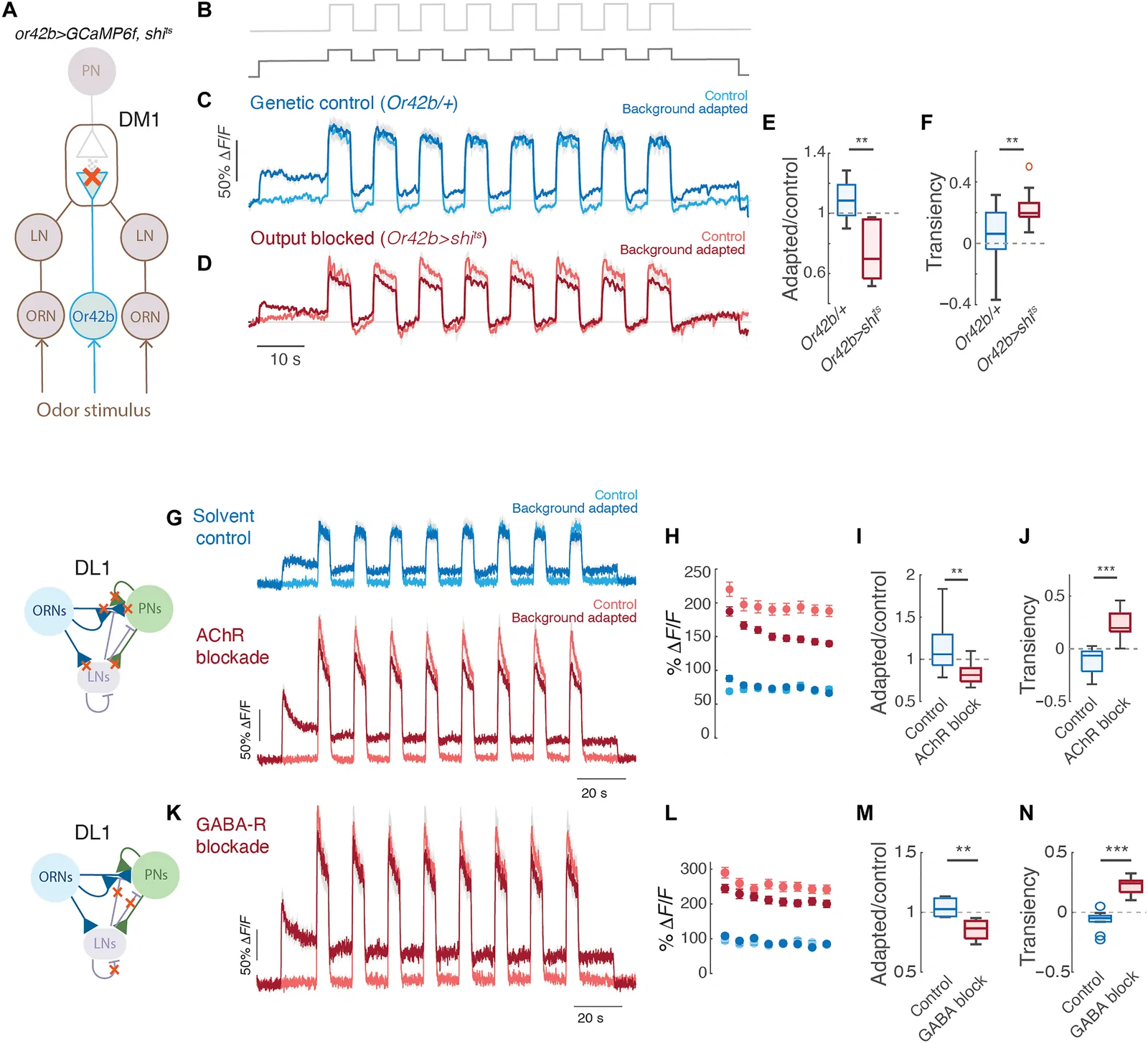
An input gain control mechanism keeps ORN presynaptic calcium background-invariant. (**A**) Genetic blockade of neurotransmitter release from Or42b-ORNs. (**B**) Stimulation protocol in control (light gray) and background-adapted conditions (dark gray). (**C**) Calcium response of the DM1 glomerulus in genetic control flies showing background-invariant responses and (**D**) calcium response in flies with blocked ORN synapses showing background-dependent responses to methyl acetate (liquid dilution of 10^−4^, background airflow dilution C5 and pulse C6; see table S3). (**E**) Relative adaptation measure as the response ratio in adapted and control conditions. (**F**) Response transiency measured as the relative change in response at test stimulus onset and offset (transiency = 1 − *f*_offset_/*f*_onset_); *n* = 7 and 8. (**G**) Calcium responses from the DL1 glomerulus before and after drug application. Pure 2-butanone was used in airflow dilutions of C7 for the background and C8 for the pulse. For the blockade of AChR, 100 μM atropine + 20 μM tubocurarine + 200 nM IMI was used; *n* = 10. (**H**) Mean response to the single pulses. Error bars indicate SE. (**I** and **J**) As in (E) and (F). (**K** to **N**) as in (G) to (J) for GABA-R blockade: 25 μM CGP54626 + 5 μM PTX; *n* = 5. Thick lines indicate the means, and shaded areas indicate SE. Box plots indicate median, quartiles, min/max values, and outliers. **P < 0.01 and ***P < 0.001, Kruskal-Wallis test.

Next, we investigated the potential mechanisms that compensate the firing rate adaptation and achieve calcium background invariance. First, we asked whether ORN presynaptic calcium is regulated in a cell-intrinsic manner or through a circuit computation. To distinguish between these two cases, we expressed the temperature-sensitive dynamin mutant shibire^ts^ in Or42b-ORNs to block neurotransmitter release and measured odor responses at the same presynapses (Fig. 3A). To capture slower adaptive processes, we presented the test odor eight consecutive times (Fig. 3B). As expected, flies of the control genotype showed tonic and background-invariant responses (Fig. 3C). In contrast, blocking synaptic release led to more transient and background-dependent responses (Fig. 3, D to F), resembling the adaptive properties of firing rate responses (Fig. 1B). Similar results were obtained for the glomerulus DL1 (fig. S1, A to D). These data indicate that calcium responses in the ORN presynapses rely on a circuit-based computation to achieve background invariance. Lateral inputs from other glomeruli, activated by this odor but unaffected by the manipulation (e.g., from DM4), are not sufficient to compensate adaptation of the DM1-ORNs. Together with the optogenetic protocol, these data provide further evidence that intraglomerular activity can be sufficient for background compensation.

ORN synapses release acetylcholine. To test that cholinergic activity is required for the background compensation, we used pharmacology to block acetylcholine receptors (AChRs). This led to transient and background-dependent responses (Fig. 3, G to J) for different combinations of test and background stimuli (DL1; fig. S2, A to C), further indicating that the regulation of the presynaptic calcium is not a cell-intrinsic property and requires a synaptic feedback loop. ORNs make most of their synapses with LNs and PNs. PNs are the major output of the AL sending olfactory information to higher brain regions, yet they also have presynapses within the glomeruli (*39*, *40*). We therefore asked whether PN synaptic activity contributes to the ORN axonal compensation. Genetically silencing PN output had no effect on presynaptic calcium in ORNs, excluding an output gain control mechanism (fig. S1, E and F). LNs form a diverse population of inhibitory neurons mediating different types of gain control in the AL (*32*, *41*), but their role in the context of background adaptation remains unexplored. To test their function, we used pharmacology against γ-aminobutyric acid (GABA) receptors. Similarly to the blockade of AChRs, GABA receptor blockade disrupted background invariance and led to more transient calcium dynamics (Fig. 3, K to N, and fig. S2A). This argues in favor of a role of GABAergic LNs in tuning background adaptation in this glomerulus.

Different glomeruli vary in their sensitivity to inhibition (*32*, *42*), prompting us to ask whether inhibition is required in all glomeruli for background invariance. Similar to DL1, blocking inhibition disrupted background compensation in DM3 and rendered calcium dynamics transient (fig. S2B). DM6 calcium responses also became transient, but despite a trend, the change in background compensation was not significant (fig. S2B). In contrast, DL5 ORNs were unaffected by the blockade of either AChRs or GABA receptors and displayed an unusual slow increase in baseline calcium across pulse repetitions that was resistant to drug application (fig. S2, A and B). While some of these differences across glomeruli might be due to drug penetration, the distinct DL5 dynamics suggest the existence of complementary strategies for background invariance. Nonetheless, our data support a predominant role of inhibition in mediating a form of presynaptic compensation that counteracts firing rate adaptation.

### An amplitude-shift adaptation mechanism is required to achieve background invariance through inhibitory feedback

**Fig. 4.**
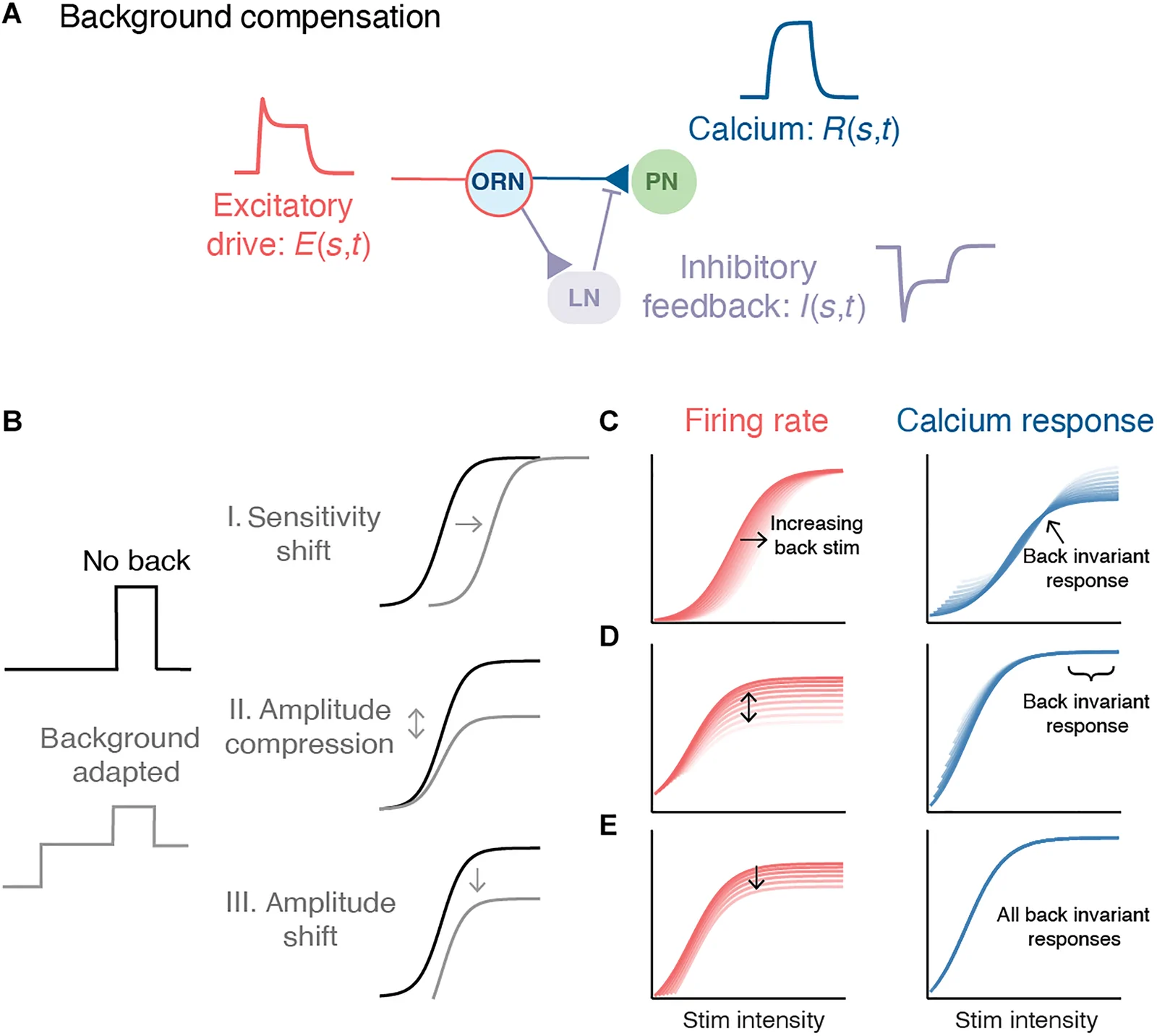
Adaptation mechanisms for background-invariant calcium response. (**A**) We consider an inhibitory presynaptic feedback loop with ORN firing rate as excitatory input (E) and ORN axonal calcium (R) as output (Materials and Methods). Sample traces are depicted. (**B**) Three types of background-induced adaptation for individual neurons, implemented as a change in a specific parameter of the ORN stimulus response curve (see Materials and Methods). (**C** to **E**) Simulated firing rate (red) and calcium response (blue) for the three different adaptive strategies: sensitivity shift, amplitude compression, amplitude shift. Different color intensities correspond to different intensities of the background stimulus.

To understand how inhibitory feedback enables background-invariant calcium responses, we developed a model linking ORN firing rates to axonal calcium dynamics. In the absence of a background, presynaptic calcium scales linearly with firing rate (Fig. 1D). We therefore modeled the presynaptic calcium response R(s,t), where *s* denotes the stimulus intensity and *t* denotes the time, as the sum of an excitatory drive E(s,t), proportional to ORN firing rate F(s), and a local inhibitory feedback I(s,t) on the presynapses: R(s,t)=a·[E(s,t)−I(s,t)] (Fig. 4A and Materials and Methods). Calcium responses are typically tonic but become transient when inhibition is blocked (Fig. 3), suggesting that inhibitory inputs are also transient, in agreement with LN transient spiking dynamics (*43*). Therefore, we model both excitation and inhibition with transient dynamics but different baselines (Materials and Methods). This ensures tonic calcium responses that, at the steady state, we can write as a linear function of the firing rate R(s)=kF(s) (Fig. 1D and Materials and Methods). Under background-adapted conditions, we modeled calcium responses as the sum of the sustained background component $F\left(s_{B}\right)$, reflecting the tonic calcium level induced by the background stimulus $s_{B}$, and an increase induced by the adapted firing rate $F_{B}\left(s\right)$, where the subscript B indicates adaptation to the background stimulus $s_{B}$. Together with the linear dependence between calcium and firing rate, this led to $R_{B}\left(s\right)=\mathrm{kF}\left(s_{B}\right)+kF_{B}\left(s\right)$, where $R_{B}\left(s\right)$ is the calcium response in background-adapted conditions. Imposing background invariance means that $R_{B}\left(s\right)=R\left(s\right)$, which yielded a linear dependency between the adapted and nonadapted firing responses

$$F_{B}\left(s\right)=F\left(s\right)-F\left(s_{B}\right)$$

This means that, to achieve background invariance through inhibitory feedback, the adaptation mechanism implemented by ORNs must act as a differential operator in the response domain, reducing the amplitude of the firing rate response curve rather than shifting it, in agreement with the decreased responses measured in ORNs (*18*, *23*).

To show the implication of this simple analytical model, we simulated ORN calcium responses for three firing rate adaptation mechanisms implemented by modifying distinct parameters of the firing rate response function (Fig. 4B and Materials and Methods): (i) sensitivity shift, the mechanism of contrast-sensitive visual neurons; (ii) amplitude compression; and (iii) amplitude shift, both consistent with ORN responses (*18*, *23*). Implementing adaptation via a sensitivity shift resulted in the greatest mismatch of calcium responses across stimulus conditions (Fig. 4C). Amplitude compression led to more background invariance at high, but not at low, stimulus intensities (Fig. 4D). As predicted, adaptation via amplitude shift was the only mechanism through which modeled calcium responses were invariant across all stimulus conditions (Fig. 4E and Materials and Methods).

To test the generality of these findings, we also implemented a stationary model of background compensation with a divisive normalization factor from adaptive LNs (fig. S3). Differently from above, this formulation does not explicitly model the firing rate and calcium dynamics, but it captures the divisive nature of the normalization implemented by LNs (*32*). As in the linear case, background invariance is guaranteed only for an adaptation mechanism that changes response amplitude, in this instance, a combination of both response shift and compression (fig. S3).

Together, these findings demonstrate that inhibitory feedback can mediate ON background invariance only if the input firing rate adapts by a change in response amplitude. This form of adaptation diverges from the efficient coding hypothesis at the single-neuron level (*2*–*4*), which predicts a shift in sensitivity, and aligns with experimental data in ORNs, revealing a different computational role for ORN adaptation.

### Odor contrast information supports invariance of odor responses across glomeruli

**Fig. 5.**
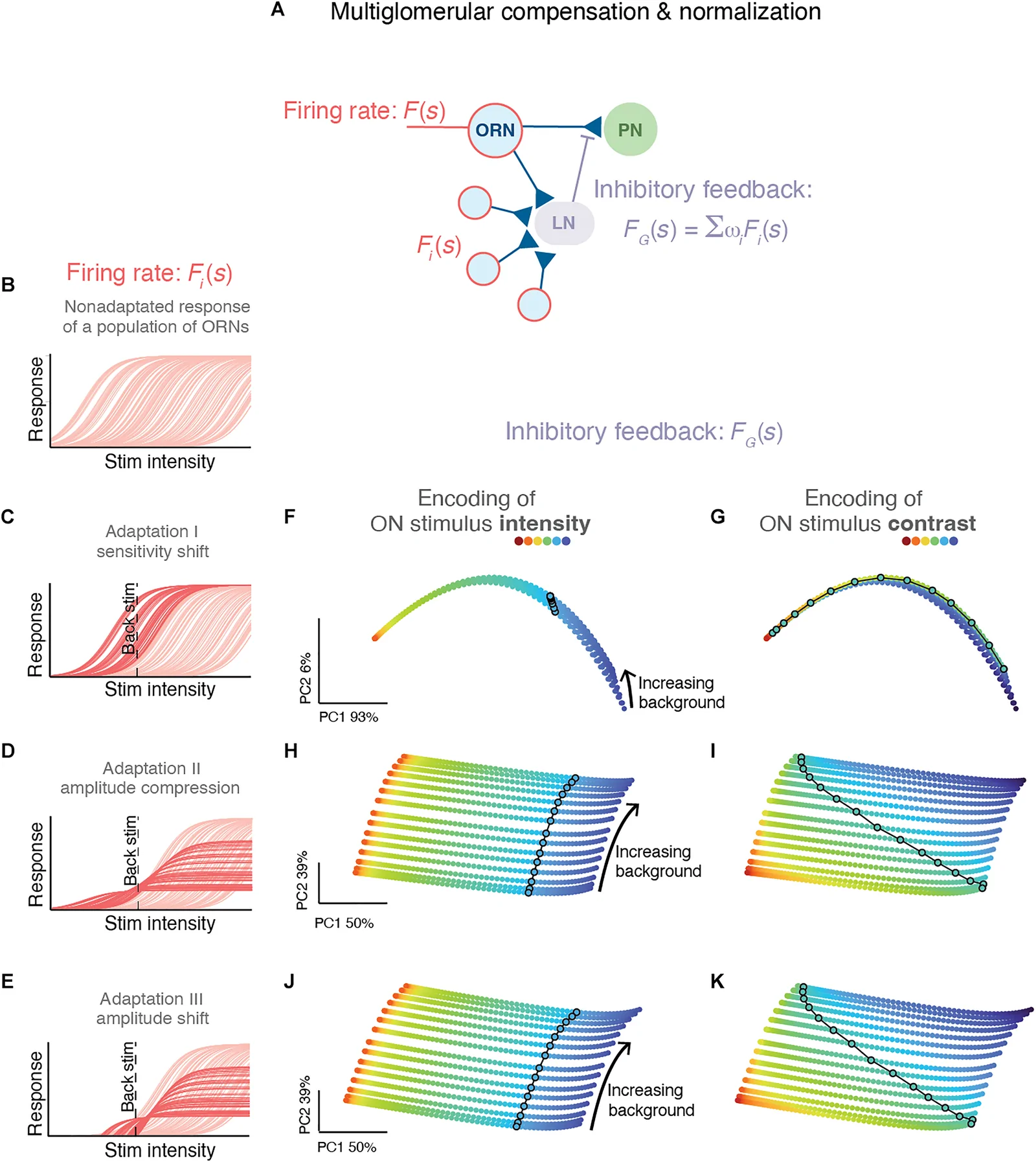
Background compensation with multiglomerular inputs. (**A**) Schematics of the circuit model that implements presynaptic compensation of firing rate adaptation, with an inhibitory term proportional to the weighted sum of the activity of all glomeruli. (**B**) Simulated stimulus response curves for a population of ORNs with different stimulus sensitivities (stimulus in log scale). (**C** to **E**) Response curves of the same neurons as in (B) after adaptation to a background stimulus indicated by the dotted line. Adaptation of the ORNs activated by the background stimulus induces a shift in sensitivity (C), an amplitude compression (D), or an amplitude shift (E). (**F**) A two-dimensional manifold of the population responses to stimuli of different intensities (color coded) in different background-adapted conditions. Black circles highlight an iso-intensity response curve across all backgrounds. (**G**) Same data as in (F) but color coded by stimulus contrast. Black circles highlight an iso-contrast response curve across all backgrounds. (**H** to **K**) As in (F) and (G) but for the other two types of adaptation.

So far, we have studied a model for background invariance on the basis of intraglomerular feedback, with an inhibitory drive proportional to the local ORN firing rate. However, most LNs take inputs from multiple glomeruli, and our experimental results show that background invariance also holds for public odors that activate multiple glomeruli (Fig. 3 and fig. S2) (*37*). We therefore asked under which conditions inhibitory presynaptic inputs from multiglomerular LNs can support background compensation. To answer this question, we modeled the inhibitory feedback as proportional to the weighted sum of the activity of multiple ORNs/glomeruli, $F_{G}\left(s\right)=\sum_{\left\{i\in G\right\}}\omega_{i}\;F_{i}\left(s\right)$, where $F_{i}\left(s\right)$ is the firing rate corresponding to the ORN i, $\omega_{i}$ is its synaptic weight onto the LN, and the sum runs over all glomeruli G (Fig. 5A). We show that, given the linearity of this multiglomerular term, background invariance holds if the firing rate adaption satisfies $F_{\mathrm{iB}}\left(s\right)=F_{i}\left(s\right)-F_{i}\left(s_{B}\right)$ for every glomerulus i (Materials and Methods), as supported by our data for private odors. Therefore, ORN adaptation by a response shift supports background invariance to both uni- and multiglomerular stimuli.

However, what are the consequences of ORN adaptation for the inhibitory drive in the AL? In nonadapted conditions, inhibitory LNs have been shown to provide a signal that is proportional to the stimulus intensity: $F_{G}\left(s\right)∼s$ (*30*, *32*, *42*). This is consistent with the fact that LNs pool inputs across glomeruli and total ORN drive increases with stimulus intensity. Within our model, satisfying background invariance further implies that $F_{\mathrm{GB}}\left(s\right)=s-s_{B}$, i.e., in background-adapted conditions, the inhibitory feedback $F_{\mathrm{GB}}\left(s\right)$ should be proportional to the stimulus contrast $s-s_{B}$ (Materials and Methods). Therefore, contrast should also be encoded in the combinatorial input to the glomeruli. To check this prediction, we simulated the response of a population of ORNs, with each ORN modeled as in Fig. 4 but with random stimulus sensitivity [Fig. 5B; see Materials and Methods and see also (*44*)]. As with single units, ORNs that respond to the background stimulus can adapt their firing response by three mechanisms: (i) shift in sensitivity (Fig. 5C), (ii) response compression (Fig. 5D), or (iii) response shift (Fig. 5E). Across these adaptation mechanisms, we studied the odor representations in the space of ORNs/glomeruli using principal component analysis to visualize a low-dimensional representation of the population responses. If single ORNs adapt by shifting their sensitivity, the population responses for different stimulus and background intensities lie on a one-dimensional space that unfolds along the first principal component and strongly correlates with stimulus intensity (Fig. 5F). Background adaptation leads to similar representations of iso-intensity stimuli (Fig. 5F, black circles). Notably, the encoding of contrast information in this population is ambiguous, as the same contrast can be represented by very different configurations (Fig. 5G, black circles). On the contrary, in populations of neurons that adapt their response amplitude by a compression or a shift, odor representations lay on a two-dimensional manifold, allowing the parallel encoding of both intensity (Fig. 5, H and J) and contrast (Fig. 5, I and K). More intuitively, when adaptation induces a shift in sensitivity, the total amount of activity generated by the ORN population is the same across background conditions. Instead, when adaptation leads to a change in response amplitude, the total activity in the neural space decreases with background intensity. This shifts the representational space, creating a dimension to encode contrast as the combination of background and stimulus intensity. These results generalize across different update rules for the parameters of the ORN response curves (Materials and Methods).

Therefore, adaptation strategies at the single-neuron level determine how odor information is represented by the population. Adaptation by an amplitude shift ensures the simultaneous encoding of both contrast and intensity at the population level. LNs could therefore extract contrast information by pooling activity across glomeruli. This inhibition ultimately ensures asymmetric background invariance in ORN calcium responses for both private and public odors. This model therefore generalizes the proposed normative role of LNs in the absence of a background (*30*, *32*) to a compensatory role in background-adapted conditions.

### Asymmetric background-invariant representations are preserved in PNs

**Fig. 6.**
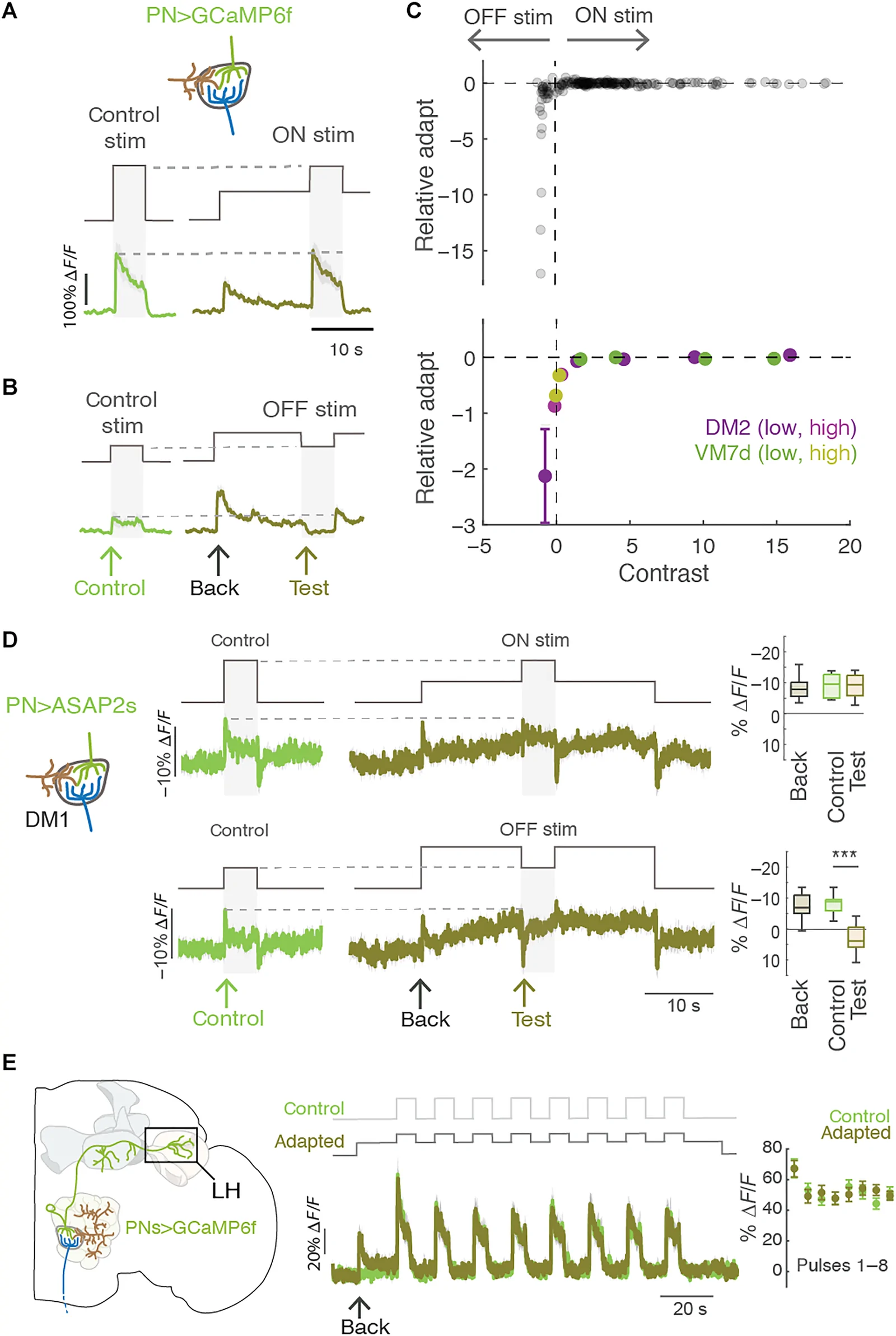
Asymmetric background invariance of PN responses. (**A** and **B**) Schematics of the stimulus protocol and calcium responses of PNs within glomerulus DM2 for two examples of ON and OFF stimuli. Colored lines indicate the mean Δ*F*/*F*, and the shaded area indicates SE; *n* = 9 to 11. The arrows indicate the control pulse (*F*_control_; green), background (*F*_back_; black), and test (background-adapted) pulse responses (*F*_test_; olive) used to define the contrast stimulus and relative adaptation. (**C**) Top: Relative adaptation as a function of contrast for each single trial, including two independent datasets recorded from two different glomeruli. Relative adaptation is defined as (*F*_test_ − *F*_control_)/*F*_control_. Given that stimulus concentrations are too low to be measured, we use response contrast as a proxy for stimulus contrast (*F*_test_ − *F*_back_)/*F*_back_ under the assumption that the response is monotonic. Bottom: Relative adaptation averaged in bins of contrast, color coded by glomerulus type. Low and high indicate two different datasets where different ranges of background concentration where used (*n* = 9 to 11; see also fig. S4). (**D**) Control and background-adapted responses measured in DM1 using the voltage sensor ASAP2s. Thick lines indicate the mean Δ*F*/*F*, and the shaded areas indicate SE. Liquid dilution of methyl acetate is 10^−2^, and air flow dilutions are B6C9 for ON stimuli and B9C6 for OFF stimuli; *n* = 10. Box plots represent median, quartiles, min/max values, and outliers, ***P < 0.001, Kruskal-Wallis test. (**E**) Calcium responses measured from the PN axons in the LH; *n* = 6. Odor stimuli were chosen to excite a single glomerulus (DM4; B5C5 with 10^−6^ and 10^−4^ liquid dilutions).

To understand the relevance of ORN axonal computations, we investigated background adaptation in postsynaptic neurons. ORNs make most of their synapses on uniglomerular PNs. ORN-PN synapses undergo depression on both fast (*28*, *43*) and slow (*37*) timescales. We reasoned that even a tonic activation of depressing synapses would result in phasic and adaptive responses in postsynaptic neurons. Contrary to ORNs, PN calcium dynamics are phasic [as also shown in (*37*)], but, unexpectedly, their responses to odors are independent of background adaptation, as in the ORN axons (Fig. 6A).

To validate the generality of this observation, we explored a range of ON and OFF stimuli, i.e., odor pulses higher or lower than the adapting background stimulus (Fig. 6, A and B). PN responses to ON stimuli had similar amplitudes independently of background adaptation (Fig. 6A and fig. S4A), also for high background concentrations (fig. S4B). In contrast, OFF stimuli, even of small amplitude, always produced large decreases in calcium (Fig. 6B and fig. S4, B and C). To capture the transition between ON and OFF responses irrespective of experimental noise in the generation of the stimuli, we quantified relative adaptation as a function of contrast in single trials. We use response contrast (defined as the relative difference between the response to the control pulse and the response to the background onset) as a proxy for stimulus contrast and define relative adaptation as the relative difference in the response to the pulse between control and adapted conditions (defined in fig. S4C). Zero contrast means that the tested stimulus equals the background, and zero adaptation means that the PN response is background-invariant. This single trial analysis captured the sharp transition between ON and OFF stimuli (Fig. 6C), demonstrating that, similarly to ORN axons, background invariance holds also in the PN dendrites only for ON stimuli. Responses to OFF stimuli instead fell much below the nonadapted response, even for very small contrast values. Therefore, the asymmetric background invariance of ORN axonal calcium is preserved in PNs.

These observations contrast with a previous study that described background adaptation in PN firing rates (*45*). This could be attributed to a more precise control of test and background stimuli in our experimental setup (fig. S4, F and G). However, it could be also due to the different measures of neural activity: calcium versus voltage. PNs’ subthreshold potentials and firing rates follow a linear relationship (*45*); therefore, we performed optical voltage recordings from PN dendrites. Using the voltage sensor ASAP2s (*46*), we show that PN dendrites depolarize similarly (Fig. 6D, DM1) or even slightly more (rather than less; fig. S4, D and E, DM2 and VM7d) when odor stimuli are presented on a background (ON stimuli). Consistently with calcium measurements, OFF responses are not background-invariant (Fig. 6D). Last, we asked whether these properties are preserved in the PN output. Calcium imaging from the axonal innervations of PNs in the lateral horn (LH) shows phasic and background-invariant odor responses (Fig. 6E), further supporting the relevance of these dynamics for information transmission. We conclude that both PNs’ dendritic and axonal activities encode a background-invariant representation of ON odor stimuli, overcoming the background adaptation of ORN firing rates (*17*, *18*) and the depression of ORN-PN synapses (*28*, *37*).

### PNs’ background invariance requires modulation of ORN synaptic release

**Fig. 7.**
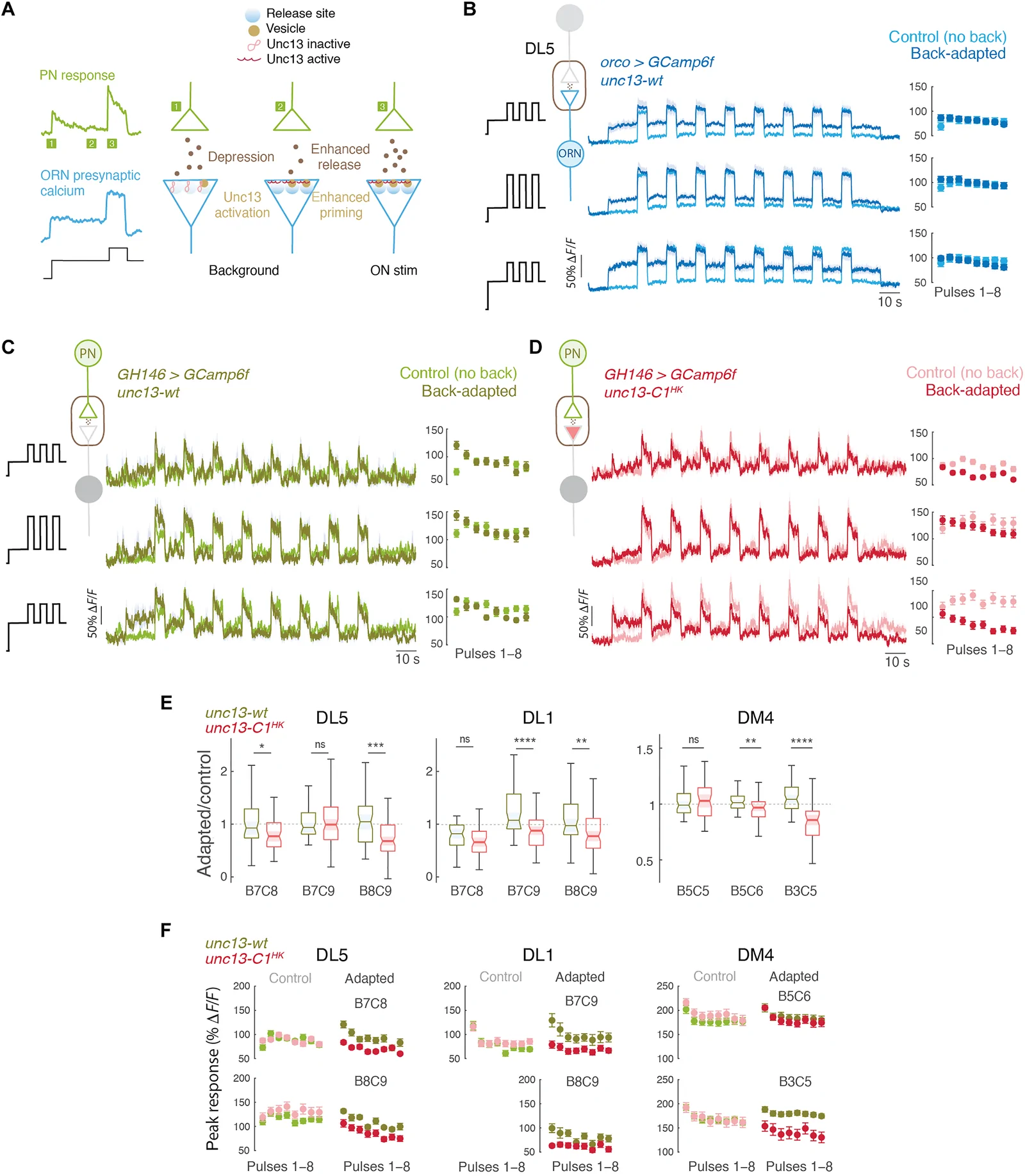
The Unc13-C1 domain is required for background-invariant PN responses. (**A**) Schematics of the hypothesis. (**B**) Mean calcium response from the ORN axon terminal to eight odor pulses in control and background-adapted conditions for three combinations of background and test stimuli. Shaded areas indicate SE. Right: Quantification of the mean peak response to each odor pulse. (**C** and **D**) Same as (B) for PNs in control and Unc13-C1^HK^ mutant flies [wild type (WT): *n* = 6 to 7; mutant: *n* = 7 to 11]. (**E**) Background-adapted response divided by the control for each stimulus condition tested in three different glomeruli. For DL5 and DL1, pure 2-butanone was used for background and pulse. For DM4, we used methyl acetate with liquid dilution of 10^−6^ for (B)and 10^−4^ for (C) (in B5C5 and B5C6) or just 10^−4^ (in B3C5). Bar plots are generated from all flies measured and all eight puffs in each trial (DL1 and DL5: control: *n* = 6 or 7; mutant: *n* = 7 to 11; DM4: control: *n* = 6 to 8; mutant: *n* = 6 or 7). Bar plots indicate median, notch, quartiles, and max and min. BXCX indicates the flow rate for background (B) and test (C) stimuli; see table S3. *P_corr_ < 0.05, **P_corr_ < 0.01, ***P_corr_ < 0.001, ****P_corr_ < 0.0001, Kruskal-Wallis test. *P* values are corrected for multiple comparisons. (**F**) Mean peak response for control and Unc13-C1^HK^ mutant flies for eight consecutive odor puffs in control and background-adapted conditions. Error bars indicate SE.

Last, we asked how the depressing ORN-PN synapses could preserve background invariance. One key difference between calcium signals from ORN presynapses and PN postsynapses is their dynamics: tonic in ORNs and phasic in PNs. We reasoned that tonic levels of presynaptic calcium—induced by the sustained background—might be associated with enhanced engagement of the neurotransmitter release machinery (Fig. 7A). Conserved Unc13 proteins mediate several types of presynaptic plasticity (e.g., short-term facilitation and fast and long-term potentiation) (*47*–*54*). The two Unc13 splice variants, Unc13A and Unc13B, are differentially organized at the ORN-PN synapses and mediate different postsynaptic currents (*55*), but in which context they mediate plasticity at ORN-PN synapses remains unclear. Unc13-dependent plasticity has been linked to three domains that can be modulated directly or indirectly by calcium: the Ca^2+^/calmodulin (CaM) binding domain (only present in Unc13A), the C1 domain binding diacylglycerol (whose metabolism is Ca^2+^-dependent), and the C2B domain, which binds phosphoinositides in a Ca^2+^-dependent manner (*47*, *56*). The C1 and C2B domains were shown to exert autoinhibition on the vesicle priming reaction, which makes vesicles responsive to action potentials (*57*). Activation of the Unc13-C1 domain enhances action potential–induced transmitter release and diminishes multiple forms of presynaptic plasticity (i.e., short-term facilitation, posttetanic potentiation, and homeostatic potentiation) (*54*, *58*–*60*). A single amino acid exchange (H→K) in the C1 domain reconstitutes this enhanced potentiation and blocks synaptic plasticity (short-term facilitation and potentiation) (*54*, *59*, *61*). We used the Unc13-C1^HK^ mutation to test whether ORN presynaptic plasticity is required to achieve background invariance. We used three stimulus conditions combining two concentrations of the background and test stimuli, which, in control flies, led to background-invariant responses in both DL5 ORNs and PNs (Fig. 7, B and C). It should be noted that, in this glomerulus, the background compensation is initially overestimated, and background invariance is achieved only at the second odor pulse. Such invariance was lost in Unc13-C1^HK^ mutants for two of the three stimulus conditions tested (Fig. 7D). We did not see major effects of the C1 domain mutation for the high contrast condition, which indicates that the calcium modulation probably involves more than one mechanism. Comparing control (wild type) and mutant flies across three different glomeruli (including DL1 and DM4), we observed significant effects of the mutation on relative adaptation for most stimulus conditions tested (Fig. 7E). Unlike what would be expected from a loss of function of Unc13 in ORNs (*55*), the C1 gain-of-function point mutation had no effects on the response to odor stimuli delivered in isolation and specifically affected only the response in background-adapted conditions (Fig. 7F). Therefore, (*54*) only activity-dependent modulation of the domain is disrupted, in agreement with measurements at the larva neuromuscular junction (54), while synaptic transmission itself is not impaired or attenuated in this context.

Overall, we conclude that modulation of Unc13 proteins is necessary to keep the PN postsynaptic response background-invariant and stable over time. This pathway might act redundantly or synergistically with other calcium-dependent modulations of presynaptic function.

### A population code with asymmetric background invariance

**Fig. 8.**
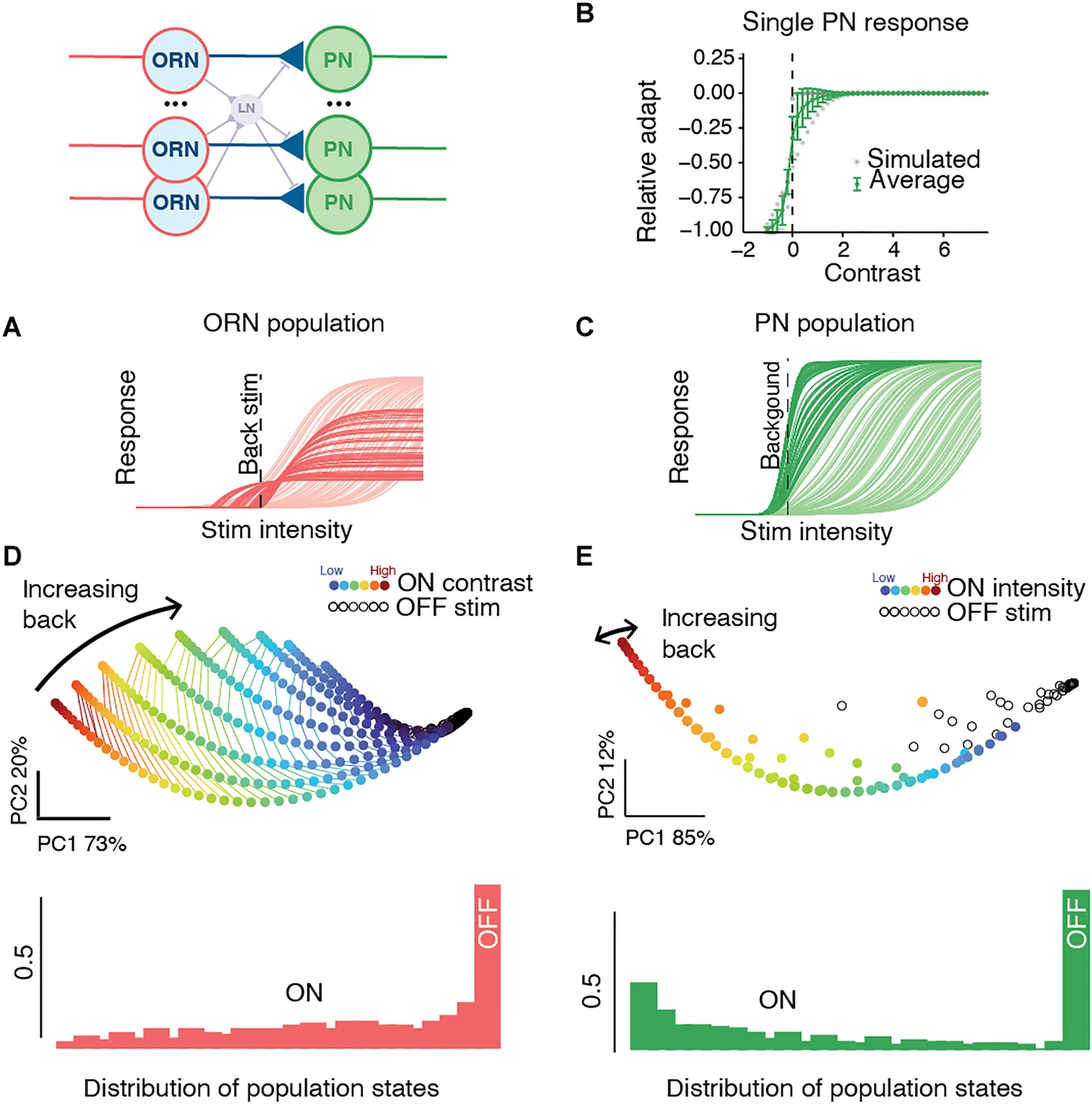
Combinatorial representation of ON and OFF stimuli in ORN and PN populations. (**A**) Firing rate response of 100 ORN types adapted to the background stimulus, indicated by the dashed line. (**B**) Model of PN adaptation as a function of contrast. The transition from negative to positive contrast is modeled as a noisy process with standard deviation σ=0.5 (see Materials and Methods). (**C**) Response of 100 PN types adapted to the background stimulus indicated by the dashed line. (**D**) Two-dimensional representation of ORN population response to stimuli of different ON (color coded) and OFF (black) contrast in different background-adapted conditions (arrow). The histogram represents the distribution of population states calculated on the first principal component. (**E**) Same as (D) for PNs, but the color code represents stimulus intensity. Note that many responses to OFF stimuli are not visible as they overlap in a small region.

So far, we have shown that ORN firing rate adaptation by a response shift is required for background compensation of presynaptic calcium mediated by inhibitory presynaptic feedback, ultimately leading to PNs’ asymmetric background invariance. However, why is ORN firing rate adaptation undone and why asymmetrically for ON and OFF stimuli? To address this question, we compare how odor information for ON and OFF stimuli is represented in a population of ORNs and PNs. We model individual ORN firing rates using the amplitude-shift model of adaptation with the update rule that ensures background invariance (Fig. 8A and Eq. 14 in Materials and Methods). This is equivalent to Fig. 5J, but now, we also consider OFF stimuli. We model individual PNs as background-invariant using Gaussian noise to recapitulate the sharp transition between ON and OFF stimuli, which we attribute to the low signal-to-noise ratio in their input (Fig. 8, B and C, and Materials and Methods). As shown for ON stimuli only, a population of ORNs encodes contrast information explicitly, but the odor representations shrink close to zero contrast, leading to a continuous transition between ON and OFF stimuli (Fig. 8D). On the contrary, the response space of the PN population exhibits a discontinuity that separates ON and OFF stimuli across different backgrounds while preserving a background-invariant representation of ON stimuli (Fig. 8E). Overall, we propose that the AL implements a circuit computation that extracts contrast information encoded in ORNs to enhance the discriminability between ON and OFF stimuli in postsynaptic PNs while preserving information of odor identity for ON stimuli.

## DISCUSSION

Odors are detected by single sensory neurons that work combinatorially to represent complex natural stimuli at the population level. Despite the key role of adaptation for information coding in both single neurons and neuronal populations, the adaptive properties of the olfactory system downstream of the receptors have been largely overlooked. In this work, we investigated adaptation as a coding strategy of single ORNs that shapes the encoding capabilities of olfactory neural populations. By combining experiments and computational models, we identified a property of the olfactory pathway that we defined as asymmetric background invariance. We show that the adapted firing responses of single ORNs are compensated to achieve an asymmetric representation of ON and OFF contrast stimuli, where ON responses are background-invariant, while OFF responses collapse in the representation space of the downstream populations, the PNs. Mechanistically, background invariance requires a feedback compensation induced by ORN synaptic release and involves a GABAergic inhibitory pathway that modulates ORN presynapses. We propose that this feedback compensation facilitates vesicle release through an interaction with the Unc13-C1 domain, which stabilizes the background invariance of the PNs. The PN population therefore preserves the ON stimulus identity necessary for downstream computations.

### Role of adaptation at the single-neuron and population level

Efficient coding principles provide an explanation for how single sensory neurons adapt their response function to changes in stimulus statistics over behaviorally (*12*, *13*, *62*) or evolutionarily (*63*–*65*) relevant timescales. Similar principles have been used to understand how the response function of neurons in a population should adjust to tile the stimulus space in a flexible manner (*66*). This is of particular importance to understand coding principles in the olfactory system, which uses a repertoire of receptors to detect and discriminate odors within neuronal populations. Receptor repertoires are tuned, through evolutionary times, to the ecology of the animal (*67*), leading to efficient sensory codes in the ecologically relevant stimulus space (*68*). A similar adaptive strategy also applies on shorter timescales. For instance, the expression levels of the receptors are plastic in mammals, providing a mechanism to optimize the composition of a receptor repertoire to changes in the environment (*69*).

Similar adaptive changes in the coding capability of a receptor repertoire could in principle occur also on even shorter timescales, but there is limited evidence of such a strategy in the olfactory system. In this work, we provided evidence that the fly olfactory system does not adapt the odor representation to the background. Rather, it implements mechanisms that keep odor representations robust with respect to changes in the background. While efficient coding theories of single neurons predict the conventional sensitivity shift widely observed in the visual system, both ORNs and PNs do not follow this principle. ORNs adapt by modifying their response amplitude (and not their sensitivity). We suggest that this allows the compensation necessary to achieve asymmetric background invariance in downstream neurons, the PNs. Therefore, even though a persistent stimulus (the background) induces synaptic depression and a corresponding decrease in postsynaptic activity (*28*, *37*, *43*, *70*), it also primes the synapses to respond to ON stimuli as strongly as in the absence of a background.

Our findings raise the question of why ORNs would adapt at all if the goal was to encode absolute stimulus intensities. We argue that firing rate adaptation is beneficial to the asymmetric encoding of ON and OFF stimuli, where only ON stimuli preserve stimulus identity, while OFF stimuli collapse into a low-response state. Such asymmetry would not be possible if the ORNs simply encoded stimulus intensity. From a more speculative perspective, another potential benefit of adapting firing rate and then compensating it downstream could be a lower total energetic cost of sending information through the brain with no loss. However, further work is necessary to assess whether the synaptic computations that compensate firing rate adaptation are less energetically costly than implementing a nonadaptive spiking code.

### Role of asymmetric odor encoding downstream of PNs

PNs send odor information to both the LH and mushroom body (MB), contributing to higher-order computations. Odor representations in the Kenyon cells (KCs) of the MB are used to form associative memories on the basis of the co-occurrence of an odor and a reinforcement (*71*). For memory retrieval, it is critical that the same KCs are activated by the conditioned stimulus in training and test phases, and, therefore, the odor must be represented independently of possible backgrounds. Thus, memory retrieval provides a potential function for background-invariant encoding in PNs. Consistently with this hypothesis, PNs in the locusts maintain an adaptation-invariant representation of stimulus intensity, which can aid stimulus discrimination (*72*).

Odor cues are used not only to evaluate but also to explore the environment. Animals use odor gradients (*73*, *74*), plume statistics (*75*, *76*), and odor motion (*77*) to direct their behavior in complex olfactory environments. These computations require the detection of gradients and contrast in an odor-specific manner. It is plausible that such computations do not occur in the AL, where odor representations are formed, but in downstream neurons that can read out a population response from the AL to then compute stimulus-specific information on contrast. The LH provides the cellular complexity (*78*, *79*) to perform odor-specific computations in parallel (*80*), including the encoding of contrast (*81*). We suggest that the encoding of background-invariant ON responses through the compensation of ORN adaptation is essential for downstream stimulus-specific computations that drive key behaviors in flies. These computational requirements do not generalize to OFF stimuli, which have a very different relevance for the fly behavior compared to ON stimuli, leading to an explorative search rather than an oriented behavior (*73*, *82*).

### Cell-intrinsic and circuit mechanisms that maintain ON stimulus invariance

Asymmetric background invariance is the most robust observation we made across all our measurements in both ORN axons and PN dendrites. However, we also encountered differences across glomeruli. First, ORN calcium dynamics range from being slightly phasic (DM1), completely flat (DL1) to slightly ramping up (DL5). This seems to correlate with when background invariance is achieved: already on the first (DM1) or only on the second or third (DL1, DL5) odor pulse. Moreover, we showed that the background invariance of DM1 and DL1 ORNs requires synaptic transmission, whereas DL5 background invariance does not. Furthermore, blocking GABA receptors has differential effects across glomeruli, which is unlikely to be explained only by differences in drug penetration. Glomeruli such as DL5, which do not receive substantial GABAergic inputs under the conditions tested, may instead rely on cell-intrinsic mechanisms—for example, the regulation of internal calcium storages (*83*, *84*). In other glomeruli, these intrinsic mechanisms may act in combination with feedback inhibition, which is recruited in a stimulus-dependent manner.

In all tested glomeruli, we find that a gain-of-function mutation of the Unc13-C1 domain, Unc13-C1^HK^, occludes PN background invariance. So far, we do not have direct evidence that plasticity involving Unc13 is mediated by the tonic and background-invariant presynaptic calcium nor that it is downstream of lateral inhibition. However, our current working model postulates a link between presynaptic calcium regulation and enhanced vesicle priming. As the Unc13-C1^HK^ mutant does not abolish background invariance for all stimulus conditions, other calcium-dependent mechanisms are likely involved in this plasticity, perhaps through modulation of other synaptic proteins [e.g., synaptotagmin-3 or synaptotagmin-7 (*85*)].

Overall, our data suggest a key role for presynaptic plasticity in PN background invariance, linking the same molecular pathways usually studied under artificially induced homeostasis (*47*, *54*, *86*) to sensory computations in natural conditions. At ORN-PN synapses, the homeostatic set point is the nonadapted response. Differently from other models of synaptic homeostasis, the feedback compensation is not calculated on the basis of synaptic output (PN response) but on the basis of the input drive to synaptic release (ORN firing rate). This synaptic strategy is, to our knowledge, unique in its design and perhaps specific to the computational demands of the olfactory system.

### Undoing of a neural computation: Evolutionary considerations

Undoing an upstream computation is rather an uncommon observation that becomes counterintuitive from an efficient coding perspective, where computations are expected to optimize information processing across neuronal layers. However, if we look at neural circuits as ongoing projects of evolution that continue to adapt to optimize behaviorally relevant trade-offs (*87*, *88*), more examples of undoing can be found. Some amacrine cells in the visual system undo the color opponency calculated in upstream neurons to preserve the balance between opponent and non-opponent units in the retina (*89*) such that both types of information are transferred to downstream neurons. The logic of this computation could result from the order in which these mechanisms evolved [discussed in (*89*)].

From an evolutionary perspective, the olfactory system is designed to ensure adaptability, which is implemented primarily by the evolution of chemosensory genes. Our considerations focused on the combinatorial aspect of odor coding and, therefore, the fact that stimuli are encoded in populations of sensory neurons, with identity defined by the odorant receptor (OR) they express. However, this system evolved from an ancestor that expressed only one or few ORs (*90*). In such a low-dimensional system, firing rate adaptation might not only have been parsimonious but also an efficient strategy for odor encoding, as it is the case for visual neurons. The olfactory system might have selected for mechanisms that support background invariance only after the emergence of a combinatorial coding strategy with multiple ORs.

Olfactory systems also evolved specialized channels that encode unique chemicals, such as pheromones (*91*) or harmful substrates (*92*), crucial in certain ecological contexts. These stimuli therefore do not require a population code and might implement different strategies for adaptation. A combination of mechanisms can be therefore expected within the same sensory system depending on the function of specific subcircuits. Further insight into these questions might benefit from the study of downstream areas like the LH that read out combinatorial or specialized representations for behaviorally-relevant computations.

## MATERIALS AND METHODS

### Experimental model/fly husbandry

All flies were kept on a standard molasses-based food at 25°C and on a 12-hour:12-hour light-dark cycle or at room temperature in the laboratory. For optogenetic experiments, flies were kept in the dark and on food that was supplemented with 1 mM all-trans retinal (Sigma-Aldrich; table S1) for at least 4 days before experiments. For shibire^ts^ experiments, flies were incubated for at least 1 hour at 36° to 37°C before the start of recordings, which lasted a maximum of 45 min. Female flies were used for all experiments. The complete genotypes are given in table S2.

### Fly mutagenesis of Unc13-C1^HK^

The mutation of the Unc13-C1 domain was based on the corresponding mutation of the mouse homologs Munc-13-1 where histidine-567 was changed to lysine (*59*). Sequence alignment with *Drosophila* Unc13A identified histidine-1723 as the relevant target. Note that the two *Drosophila* splice isoforms Unc13A and Unc13B harbor a C1 domain (*53*) and that this modification therefore affects both. CRISPR-mediated mutagenesis was performed by WellGenetics Inc. using modified methods of Kondo and Ueda (*93*) to generate a break in unc-13/CG2999. A PBacDsRed cassette containing two PBac terminals and 3xP3-DsRed and two homology arms with point mutations (one to induce the amino acid change and one to introduce a TTAA sequence to recognize proper integration on the other homology arm) were used for repair. The DsRed marker was intermittently used to identify flies with successful integration events in the F1 generation. The marker was later excised, and the presence of the point mutation and successful removal of the cassette in the final fly line were validated by polymerase chain reaction and sequencing from genetic DNA.

### In vivo calcium imaging

Flies 6 to 10 days posteclosion were anesthetized on ice and mounted on a custom holder using the Bondic repair system [Bondic cartridge with Bondic liquid plastic and Bondic ultraviolet light-emitting diode (LED)]. The proboscis was secured, and the maxillary palps were covered using low-melting-temperature wax. A saline solution (5 mM Hepes, 130 mM NaCl, 5 mM KCl, 2 mM MgCl_2_, 2 mM CaCl_2_, and 36 mM saccharose, pH 7.3) was added, and the cuticle covering the fly’s brain, as well as obstructing trachea and fat tissue, was removed.

Functional imaging was performed on an Investigator two-photon microscope (Bruker) coupled to a tunable laser (Spectraphysics Insight DS+) with a 25×/1.1 water-immersion objective (Nikon). Laser excitation was tuned to 920 nm, and less than 20 mW of excitation was delivered to the specimen. Emitted light passed through an SP680 short-pass filter, a 560 lpxr dichroic filter, and a 525/70 filter. Photomultiplier tube gain was set to 850 V. The microscope was controlled with PrairieView (5.4) software. GCaMP6f was used for all calcium imaging experiments—please note that data in Fig. 1 (C and D) were originally collected using GCaMP3 by Martelli and Fiala (*37*).

### Stimulation

Test stimuli (pulses) were 5 s long, and background stimuli were 15 s. In Figs. 4 to 6, test stimuli were repeated eight times with 10 s between repetitions.

#### Optogenetics

Light from a 625-nm LED was directed to the fly’s antenna using an optic fiber. In calcium imaging experiments, the LED was controlled by the imaging software and activated during the laser flight-back, allowing simultaneous acquisition and excitation. In electrophysiological experiments, the LED was controlled by an Arduino board. The slightly shifted range of light intensities is because firing rate responses saturated already at 0.4 μW/mm^2^. Therefore, when we moved on to performing calcium imaging experiments, we decided to sample a lower range of intensities to avoid the saturated regime.

#### Odor delivery

In calcium imaging experiments, a flow of clean air was presented to the fly continuously (1 liter/min). To this main airflow, either an odor (odorant airflow; 100 ml/min) or more clean air (balancer airflow; 100 ml/min) was added through a solenoid valve (LEE) so that the final airflow reaching the fly was always around 1.1 liters/min. To create the gas dilutions for the odorants, four mass flow controllers were used (Analyt-MTC) and controlled using a custom MATLAB (MathWorks) script and an Arduino board. Another three mass flow controllers were used to keep the constant airflows for the main airflow and the balancer airflows. Odors were prepared in 20-ml glass vials as a liquid volumetric dilution with a total volume of 5 ml in mineral oil. Airflow dilutions used are described in table S3. 

### Electrophysiology

Single-sensillum recordings were performed as previously described (*18*) using a silver chloride electrode and glass pipettes filled with a sensillum lymph ringer. Electrical signals were amplified using an extracellular amplifier (EXT-02F-1, npi) with a head stage (EXTEH), bandpass filtered (300 to 5000 Hz), and digitized at 20 kHz using a NI board (NI-6212). Data were acquired with the MATLAB toolbox controller (https://github.com/emonetlab/kontroller) (*25*). Spikes were sorted using a custom MATLAB script. For odor delivery, flies were exposed to a constant airflow (1 liter/min), and an odor stimulus was delivered by switching a three-way solenoid valve that directed a secondary airflow (100 ml/min) through a Pasteur pipette as in (*18*, *37*). The pipette contained a filter paper with 50 ml of the odor dilution. Volumetric odor dilutions were prepared in either mineral oil or Milli-Q water. Stimuli were controlled by custom-made software in MATLAB and Arduino.

### Pharmacology

AChR blockade was performed using 100 μM atropine + 20 μM tubocurarine + 200 nM imidacloprid (IMI). Stock solutions of 2 mM atropine and 20 mM tubocurarine were prepared in water, aliquoted, and frozen at −20°C. A stock solution of 1 mM IMI was prepared in dimethyl sulfoxide (DMSO) and kept at room temperature. A final solution was dissolved in the saline solution used for in vivo calcium imaging and kept at 4°C. 

GABA receptor blockade was done using 25 μM CGP54626 + 5 μM picrotoxin (PTX). Stock solutions of 25 mM CGP54626 and 100 mM PTX were prepared in DMSO, aliquoted, and kept at −20°C. A final solution was dissolved in saline solution used for in vivo calcium imaging and kept at 4°C.

For all pharmacology experiments, flies were prepared for in vivo calcium imaging, as explained before, with the addition of a final step during dissection where the brain was desheathed. All flies were imaged before application of drug solutions with the same stimulation protocol used after drug was added in bath application.

For AChR blockade experiments, the normal saline solution was replaced with 100 μM atropine + 20 μM tubocurarine + 200 nM IMI solution two consecutive times and then again 10 min after. Recordings were started 20 min after the drug solution was initially applied. 

For GABA receptor blockade, the normal saline solution was replaced with 25 μM CGP54626 + 5 μM PTX. This was repeated 1, 2, and 10 min after this initial drug application. Recordings were started 40 min after the drug solution was initially applied.

### Background compensation model: Uniglomerular inhibitory feedback

Considering the linear dependence of calcium and firing rate responses (Fig. 1D), we model the ORN calcium R as the sum of an excitatory drive resulting from ORN firing rate and local inhibitory feedback acting on the presynapses

R(s,t)=a·[E(s,t)−I(s,t)] (1)

where a is the proportionality factor. The excitatory drive depends on the ORN firing rate response, which we model as an exponential process

$$E\left(s,t\right)=F\left(s\right)\;\left[e^{-\frac{t}{\tau_{E}}}+b_{E}\right]$$ (2)

where F(s) is the firing rate, $\tau_{E}$ is the excitatory time constant, and $b_{E}$ is the excitatory baseline. We consider the inhibitory drive to have similar dynamics ($\tau_{I}=\tau_{E}$) but a different baseline, with $b_{E}-b_{I}=\Delta_{I}=0.05$. Note that, here, we do not explicitly model the LN firing dynamics but a general inhibitory modulation of presynaptic activity. While LNs have been shown to have transient spiking dynamics (*43*), their morphologies and properties are very diverse (*41*, *94*, *95*). Moreover, the dynamics of the presynaptic inhibitory drive could result from the signaling cascade downstream of the receptors. Therefore, our model of inhibitory feedback should be considered as a phenomenological description, and the role of specific LNs remains to be tested.

Using Eq. 2, the calcium response of Eq. 1 becomes tonic and proportional to the firing rate response

$$R\left(s,t\right)=a\Delta_{I}F\left(s\right)=\mathrm{kF}\left(s\right)$$ (3)

Thus, at the steady state, we can write the calcium response as a linear function of firing rate, R(s)=kF(s). Under background-adapted conditions, we model calcium responses as the sum of the sustained background component and the calcium increase induced by the adapted firing rate, which, together with the linear dependence of Eq. 3, leads to

$$R_{B}\left(s\right)=\mathrm{kF}\left(s_{B}\right)+kF_{B}\left(s\right)$$ (4)

where the lower index, *B*, indicates adapted responses to the background stimulus $s_{B}$. The background invariance of ORN calcium responses implies that

$$R_{B}\left(s\right)=R\left(s\right)$$ (5)

Imposing this condition in Eq. 4, we obtain a linear relationship between the adapted and nonadapted firing responses

$$F_{B}\left(s\right)=F\left(s\right)-F\left(s_{B}\right)$$ (6)

This implies that, to support background invariance, firing rate adaptation must occur as a change in the response domain rather than in the stimulus domain.

### Background compensation model: Multiglomerular inhibitory feedback

To understand how interglomerular activity shapes the adapted responses of ORNs, we modeled calcium responses as we did in Eq. 1 but now considering the inhibitory feedback to be proportional to a weighted sum of the activity of all the glomeruli (see Fig. 5A)

$$G\left(s,t\right)=\sum_{\left\{i\in G\right\}}\omega_{i}\;F_{i}\left(s\right)\;\left[\text{exp}\left(-\frac{t}{\tau_{I}}\right)+b_{I}\right]$$ (7)

This term can be split into the contribution of uni- and multiglomeruli inputs

$$G\left(s,t\right)=I\left(s,t\right)+I_{G}\left(s,t\right)$$ (8)

where I equals the uniglomerular term of Eq. 1. The calcium equation then becomes

$$R\left(s,t\right)=a\cdot \left[E\left(s,t\right)-I\left(s,t\right)-I_{G}\left(s,t\right)\right]$$ (9)

Using the same dynamics from Eq. 2, we obtain

$$R\left(s,t\right)=a\Delta_{I}\left[F\left(s\right)-F_{G}\left(s\right)T\left(t\right)\right]$$ (10)

where $T\left(t\right)=\frac{1}{\Delta_{I}}\left[\text{exp}\left(-\frac{t}{\tau_{I}}\right)+b_{I}\right]$ is the transiency carried by the signals from all the multiglomeruli input, and $F_{G}\left(s\right)=\sum_{\left\{i\in G\right\}}\omega_{i}\;F_{i}\left(s\right)$ corresponds to the firing rate contribution. By modeling calcium responses under background-adapted conditions as before, we get the analogous version of Eq. 4 as

$$R_{B}\left(s\right)=\mathrm{kF}\left(s_{B}\right)+{\mathrm{kF}}_{B}\left(s\right)-k\left[F_{G}\left(s_{B}\right)+F_{\mathrm{GB}}\left(s\right)\right]\;T\left(t\right)$$ (11)

Imposing the background invariance of Eq. 5 then led us to

$$F_{B}\left(s\right)-F\left(s\right)+F\left(s_{B}\right)=\left[F_{\mathrm{GB}}\left(s\right)-F_{G}\left(s\right)+F_{G}\left(s_{B}\right)\right]\;T\left(t\right)$$ (12)

Given that the multiglomerular term, $F_{G}\left(s\right)$, is a linear combination of the ORN activity, this equation is satisfied if $F_{\mathrm{iB}}\left(s\right)=F_{i}\left(s\right)-F_{i}\left(s_{B}\right)$ holds for every ORN i, as supported by our data for private odors. Therefore, for both uni- and multiglomerular inputs to the LNs, feedback inhibition can support background invariance only if ORNs adapt by a response shift.

### Adaptation model: Simulation

We model the peak firing rate of individual ORNs as a function of the odor stimulus, s, as

$$F\left(s\right)=\alpha \left[\frac{\text{tanh}\left(s-\beta \right)+1}{2}-\gamma \right]$$ (13)

The parameters α, β, and γ control respectively the gain, sensitivity, and maximum amplitude of the response. The adapted firing rate, $F_{B}\left(s\right)$, is described by the same equation but different parameters, β′, α′, and γ′, which are updated in a background-dependent manner. We assume that adaptation can either change β (mechanism I, sensitivity-shift), α (mechanism II, amplitude-compression), or γ (mechanism III, amplitude-shift).

Using Eq. 13, we calculate the adaptation rules for the parameters that satisfy the calcium invariance of Eq. 6 for each type of adaptation strategy. In particular, we calculate the adapted value of the parameters that control each of the three adaptation mechanisms considered

$$\begin{array}{cc} \beta^{\prime }=s-{\text{tanh}}^{-1}\left\{2\left[\frac{F\left(s\right)-F\left(s_{B}\right)}{\alpha }+\gamma \right]-1\right\} & I.\text{sensitivity}-\text{shift}\\ \alpha^{\prime }=\alpha \left[1-\frac{F\left(s_{B}\right)}{F\left(s\right)}\right] & \text{II}.\text{amplitude}-\text{compression}\\ \gamma^{\prime }=\gamma +\frac{F\left(s_{B}\right)}{\alpha } & \text{III}.\text{amplitude}-\text{shift} \end{array}$$

Only the amplitude-shift mechanism (III) produces an adaptive response that does not depend on the stimulus s, ensuring that calcium background invariance is satisfied for every stimulus s. The other two mechanisms require information of both the adapted and nonadapted responses, which are not accessible simultaneously to the circuit (refer to Fig. 4).

To simulate a population of ORNs, we use Eq. 13 and assign a random stimulus sensitivity $\beta_{i}$ to each ORN type; α and γ are kept the same across the ORNs. The adapted firing rate, $F_{\mathrm{iB}}\left(s\right)$, is described by the same equation but different parameters, ${\beta^{\prime }}_{i}$, ${\alpha^{\prime }}_{i}$, and ${\gamma^{\prime }}_{i}$, which are updated in a background response–dependent way. For the simulations in Fig. 5, we used the update rules

$$\begin{array}{c} I.\;{\beta^{\prime }}_{i}=\beta_{i}+c_{\beta }\;F_{i}\left(s_{B}\right)\;\text{fixed}\;\alpha_{i},\gamma_{i}\\ \text{II}.\;{\alpha^{\prime }}_{i}=\alpha_{i}-c_{\alpha }\;F_{i}\left(s_{B}\right)\;\text{fixed}\;\beta_{i},\gamma_{i}\\ \text{III}.\;{\gamma^{\prime }}_{i}=\gamma_{i}+c_{\gamma }\;F_{i}\left(s_{B}\right)\;\text{fixed}\;\alpha_{i},\beta_{i} \end{array}$$ (14)

with $c_{\alpha }=c_{\gamma }=0.8$ and $c_{\beta }=1.$ Our results in Fig. 5 show that the adaptation mechanisms II and III (amplitude changes) lead to a population code whose variance is explained by two main principal components, while only one component is sufficient for mechanism I (sensitivity-shift). We tested the generality of these results for a larger set of weight parameters $c_{\beta },c_{\alpha },\;c_{\gamma }\in \left\{0.5,\;0.6,\ldots ,1.5\right\}$ for which we quantified the ratio of explained variance between the two first principal components. For each adaptation mechanism, we obtain the average ratios $r_{\beta }=15.46\pm 0.76,r_{\alpha }=1.62\pm 0.53$, and $r_{\gamma }=1.63\pm 0.51$, where the error corresponds to the standard deviation across all the different weight parameters. Ratios near to unity indicate that both principal components contribute significantly to the population response, while large ratio values indicate a predominant representation of the first principal component. We conclude that the results shown in Fig. 5 hold for different weights in the update rules of Eq. 14.

### PN model of asymmetric background invariance

PNs have similar asymmetric background-invariant responses as the ORN calcium; therefore, we model their adapted responses as for ORNs (Fig. 4). To account for the sharp but continuous transition from OFF to ON responses for small contrast stimuli (Fig. 6C), we implement a noise model for the relative adapted responses of PN neurons with a probability $Ν\left(s-s_{B},\sigma \right)$, where $s_{B}$ is the background stimulus, and σ is the noise standard deviation. For each contrast value, we average the relative adaptation over a set of 10 different background conditions ranging from −4 to 4 in the stimulus log scale. This noise model reproduces the transition from OFF to ON stimuli observed in the experiments (Fig. 6C). We then use this model to simulate the adapted population response (Fig. 8E).
