# Supplementary material for: Undoing of firing rate adaptation enables invariant population codes
Source: Sci Adv. 2026 Aug 19;12(34):eaeg3535. doi: 10.1126/sciadv.aeg3535 (PMC13488935; doi:10.1126/sciadv.aeg3535)
Supplement: Supplementary file 1 [file sciadv.aeg3535_sm.pdf]

Science Advances
AAAS

Supplementary Materials for

Undoing of firing rate adaptation enables invariant population codes

Sofia C. Brandão *et al.*

Corresponding author: Carlotta Martelli, cmartell@uni-mainz.de

Sci. Adv. **12**, eaeg3535 (2026)
DOI: 10.1126/sciadv.aeg3535

This PDF file includes:

Tables S1 to S3
Figs. S1 to S4

Supplementary Material

Table S1: Key resources

**Reagent or Resource**	**Source**	**Identifier**
Chemicals		
Methyl acetate	Sigma-Aldrich Co	Art. #45999, CAS 79-20-9
2-butanone	Carl Roth	Art. #T920.1, CAS 78-93-3
Mineral Oil	Sigma-Aldrich Co	Art. #330779, CAS 8042-47-5
CGP 54626 hydrochloride	Tocris Bioscience	Art. #1088, CAS 149184-21-4
Picrotoxin	Sigma-Aldrich Co	Art. #P1675, CAS 124-87-8
Atropine	Sigma-Aldrich Co	Art. #A0132, CAS 51-55-8
Tubocurarine	Sigma-Aldrich Co	Art. #T2379, CAS 6989-98-6
Imidacloprid	Sigma-Aldrich Co	Art. #37894, CAS 138261-41-3
All trans retinal	Sigma-Aldrich Co	Art. #R2500, CAS 116-31-4
Bondic cartridge with Bondic liquid plastic	Bondic	Art. #BONKART
Bondic UV-LED	Bondic	Art. #BONLED
Experimental Models: Organisms/Strains		
D. melanogaster: or42b-Gal4	Bloomington Drosophila Stock Center (BDSC)	RRID: BDSC_9972
D. melanogaster: or10a-Gal4	BDSC	RRID: BSDC_9944
D. melanogaster: GH146-Gal4	Gift from A. Fiala	Gift from A. Fiala
D. melanogaster: orco-Gal4	BDSC	RRID: BSDC_23292
D. melanogaster: UAS-GCaMP6f	BDSC	RRID: BSDC_42747
D. melanogaster: UAS-CsChrimson	BDSC	RRID: BSDC_82181
D. melanogaster: UAS- shibire^{ts}	Gift from M. Silies	
D. melanogaster: UAS-ASAP2s	Gift from T. Clandinin	
D. melanogaster: lexAop-GCaMP6f	BDSC	RRID: BSDC_44277
D. melanogaster: orco-LexA	Gift from H. Scholz	
Software and algorithms		
Fiji		RRID: SCR_002285
MATLAB	MathWorks	RRID: SCR_001622
Adobe Illustrator	Adobe	RRID: SCR_010279

Table S2: Genetic *D. melanogaster* strains

Name	Genotype	Figure
GH146 > GCaMP6f	*w-; GH146-Gal4/UAS-GCaMP6f ; +*	6a-c, 6e, 7c, 7e-f, S4a-d
GH146 > ASAP2s	*w+/w-; GH146-Gal4/UAS-ASAP2s ; +*	6d, S4d-e
or42b > GCaMP6f, csChrimson	*w+/w-; UAS-GCaMP6f/UAS-CsChrimson; or42b-Gal4/+*	2
or42b > GCaMP6f, shi^{ts}	*w+/w-; UAS-GCaMP6f/+; or42b-Gal4/UAS-shibire*^{ts}	3a-f
or42b > GCaMP6f (control)	*w+/w-; UAS-GCaMP6f/+; or42b-Gal4/+*	3a-f
or10a > GCaMP6f, shi^{ts}	*w-; or10a-Gal4/UAS-GCaMP6f; UAS- shibire*^{ts}*/+*	S1a-d
or10a > GCaMP6f (control)	*w-; or10a-Gal4/UAS-GCaMP6f; +*	S1a-d
GH146 >shi^{ts}*;* *orco > GCaMP6f*	*w-; GH146-Gal4/lexAop-GCaMP6f ; orco-LexA/UAS- shibire*^{ts}	S1e-f
GH146; orco > GCaMP6f (control)	*w-; GH146-Gal/lexAop-GCaMP6f ; orco-LexA/TM2*	S1e-f
orco > GCaMP6f	*w+/w-; UAS-GCaMP6f/+; orco-GAL4/+*	3g-n, 7b, S2
GH146 > GCaMP6f; Unc13H1723K	*w-; GH146-Gal4/UAS-GCaMP6f ; +; Unc13H1723K*	7d-f

Table S3: Airflow dilutions for odor

C= test stimulus B = background	Odor airflow (sccm)	Equivalent dilution
C4/B4	0.03	$10^{-4.5}$
C5/B5	0.10	10^{-4}
C6/B6	0.30	$10^{-3.5}$
C7/B7	1	10^{-3}
C8/B8	3	$10^{-2.5}$
C9/B9	10	10^{-2}

Supplementary Figures

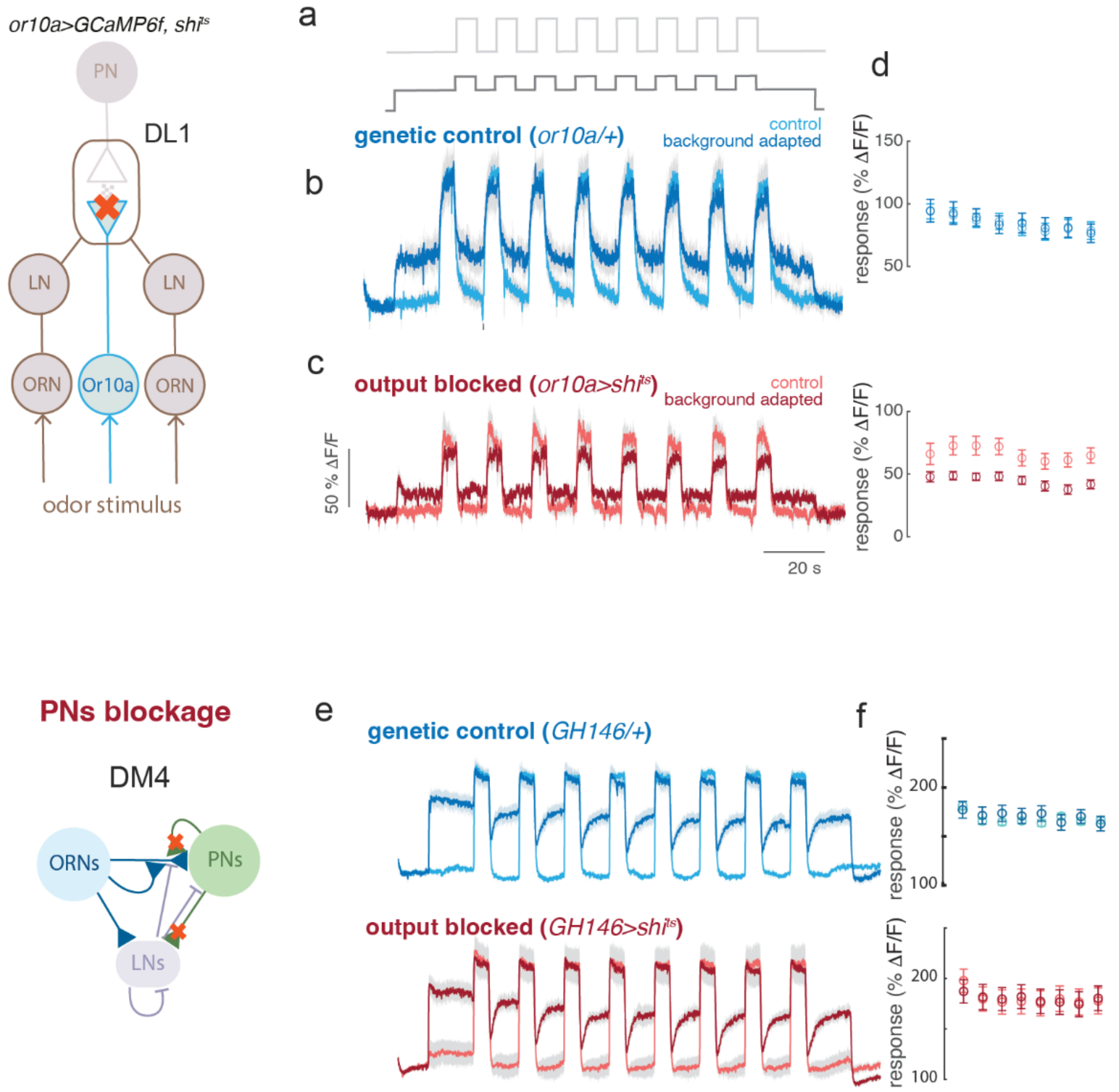

Figure S1: Role of synaptic release for ORN background invariance.
a) Genetic blockage of Or10a-ORNs neurotransmitter release. Stimulation protocol in control (light gray) and background adapted conditions (dark gray). **b)** Calcium response of the DL1 glomerulus in genetic control flies showing background-invariant responses. **c)** Calcium response in flies with blocked ORN synapses showing background dependent responses. **d)** Quantification of peak response for each of the 8 test pulses. Error bars indicate sem (N=4, 7).
e) Blocking of PN synaptic output does not affect ORN presynaptic calcium responses. Traces represent mean calcium responses of DM4 ORNs in control (light colors) and background adapted conditions (dark colors) for control (blue) and test flies (red). Test flies express shi^{ts} under control of GH146-GAL4 that labels PNs. **f)** Quantification of peak response for each of the 8 test pulses. Error bars indicate sem.

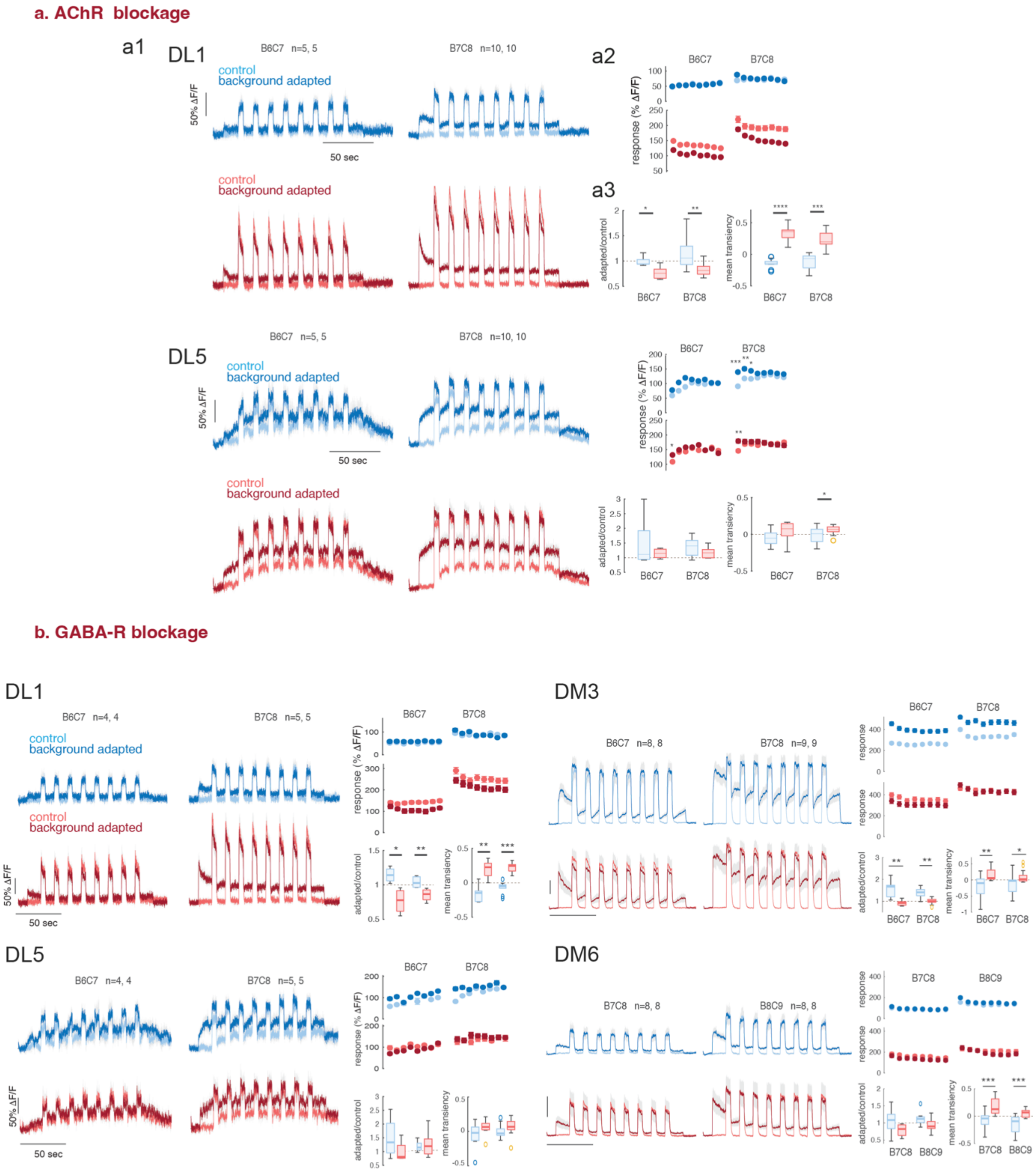

Figure S2: Glomerulus specific mechanisms for presynaptic calcium modulation.

a) Pharmacological blockage of AchRs (100 μM atropine + 20 μM tubocurarine + 200 nM imi, see Methods). **a1)** Traces represents mean calcium responses of DL1 ORNs in control (light colors) and background adapted conditions (dark colors) before (blue) and after (red) drug application. **a2)** Quantification of peak response for each of the 8 test pulses. Error bars indicate SEM but might not be visible. **a3)** Boxplot quantification of the degree of adaptation and response transiency, indicating median, quartiles max/min and outliers. **S**ame organization for glomerulus DL5.

b) Pharmacological blockage of GABA receptors (25 μM CGP54626 + 5 μM picrotoxin, see Methods) in 4 glomeruli. Same organization of the plots as in **a)**. 2-butanone was used for DL1 and DL5 and butyl acetate for DM3 (10^{-2} liquid dilution in PO) and DM6 (10^{-3} liquid dilution in PO). *p<0.05, **p<0.01, ***p<0.001. Sample size is indicated for each dataset in the figure.

IV) Amplitude compression + shift

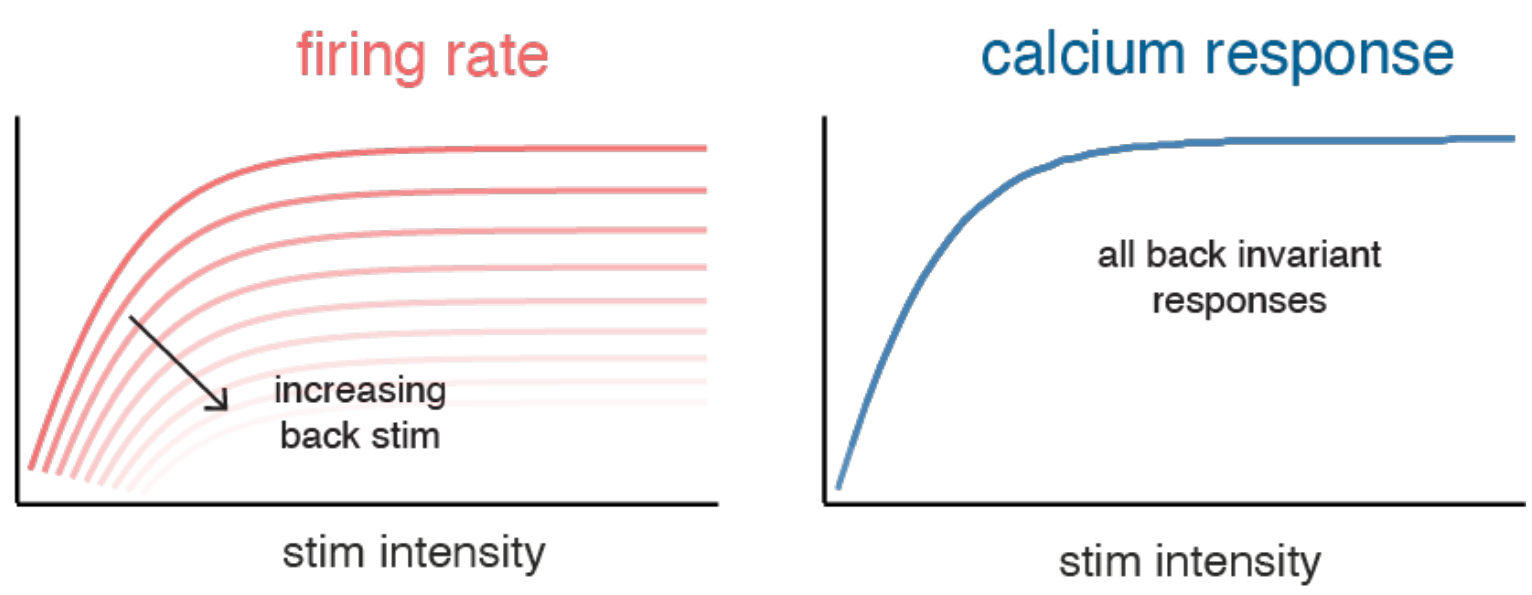

Figure S3: A divisive model of background compensation. Firing rate and calcium response for an adaptive function combining amplitude-compression and amplitude-shift as predicted by a model of background compensation and response normalization (below).

Adaptation model: In the main text we have modeled the dynamics of calcium responses in ORNs as a linear function of their firing rate and the inhibitory inputs from LNs (**Fig. 4** and Methods). However, previous work by Olsen et al.[1] has shown that LN inhibition in the AL performs a divisive (rather than linear) transformation from ORNs to PNs firing rates. Therefore, we ask if a divisive model would also be able to support the background compensation. It should be noted that the divisive model was not originally proposed to fit the transformation between ORN firing rate and ORN axonal calcium. Moreover, the model is based on mean responses and does not capture response dynamics nor considers adaptive processes. Nonetheless, in the following we show that also when assuming a divisive effect of LN activity on ORN calcium, background invariance can only be achieved if the firing rate adapts in the amplitude domain, rather than by a shift in sensitivity.

We consider the calcium response of ORNs without odor background to be:

$$R\big(F(s)\big) = \frac{F(s)}{F(s) + \sigma} \tag{s1}$$

This relationship implies that the effect of inhibition is not linear (as in Eq. 3 of the main text) but dependents on the input stimulus: $\Delta_I = 1/(F(s) + \sigma)$ with σ a constant. Background invariance is achieved if $R_B(s) = R(F(s_B)) + R(F_B(s))$ (Eq. 4 of main text). Using Eq. (s1), we obtain:

$$\frac{F_B(s)}{L(F_B(s))} = \frac{F(s)}{F(s) + \sigma} - \frac{F(s_B)}{F(s_B) + \sigma} \tag{s2}$$

where we assume a general form of the normalization factor $L(F_B(s))$ in adapted conditions. For equation s2 to hold, it is required both that:

$$F_B(s) = \sigma \cdot (F(s) - F(s_B)) \tag{s3}$$

and, using s3 in s2, that:

$$L\big(F_B(s)\big) = A \cdot F_B(s) + \sigma' \tag{s4}$$

with $A = \frac{F(S_B)+\sigma}{\sigma}$ and $\sigma' = (F(s_B) + \sigma)^2$. Eq. (s3) indicates that to achieve background invariance the adapted firing rate should be a combination of an amplitude compression (first term) and an amplitude shift (second term). In addition, Eq. (s4) indicates that background invariance with divisive normalization requires adaptation of the LN feedback and therefore a background dependent normalization term. Specifically, in adapted conditions the normalization factor is still linear but a stronger effect ($A > 1$ *and* $\sigma' > \sigma$).

Modeling a specific parametric form of $F(s)$ as in Eq.12 of the main text into Eq. (s4), we find that background invariance requires the following update rules:

$$\begin{aligned} \beta' &= \beta \\ \alpha' &= \alpha\left[1 + R_{Ca}[F(s_B)]\right] \\ \gamma' &= \gamma + \frac{\sigma R_{Ca}[F(s_B)]}{\alpha(1 + R_{Ca}[F(s_B)])} \end{aligned} \qquad (s5)$$

This shows that the combined adaptation mechanism depends uniquely on the background adaptation response in both ORNs and LNs.

1. Olsen, S. R., Bhandawat, V. & Wilson, R. I. Divisive normalization in olfactory population codes. *Neuron* 66, 287–299 (2010).

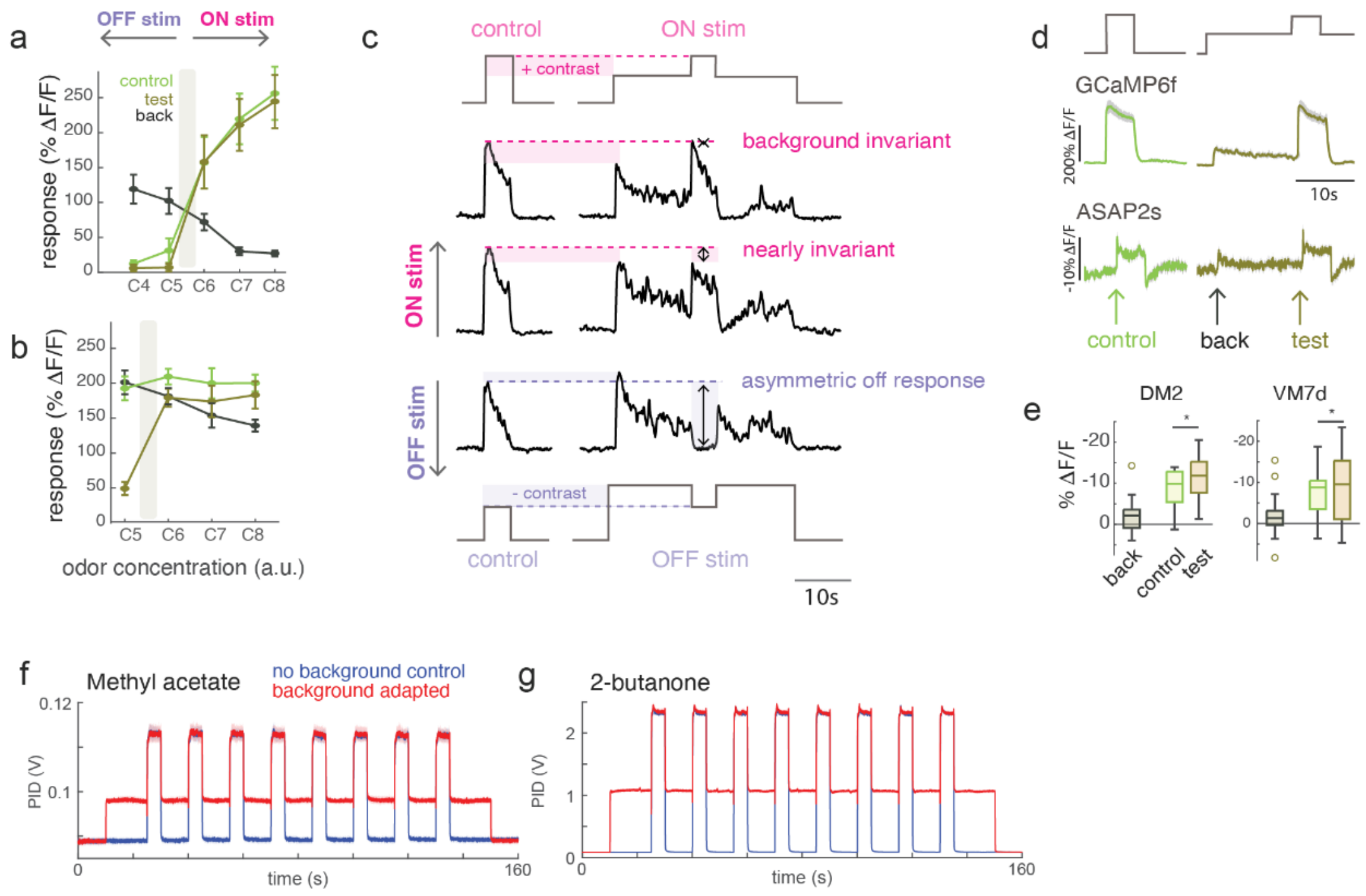

Figure S4: PNs response to ON and OFF stimuli. To cover a range of ON and OFF responses of PNs, we conducted two sets of experiments using different liquid solutions of the odors. To generate main **Fig. 6c** we analyzed the single trials of these two datasets. Here we show mean responses (**a, b**) and some examples of response to small contrast stimuli (**c**).

a-b) Peak calcium response of DM2 to different combinations of ON, OFF and background stimuli using methyl acetate. *Green*: mean response to non-adapted control. *Black*: mean response to background stimulus. *Dark green*: mean response to the same stimulus as in green after 15 seconds of background adaptation. Error bars indicate SEM, the shaded area indicated the stimulus that equals the background concentration. Liquid dilution of methyl acetate for the background was 10^{-4} in **a)** and 10^{-3} in **b)**, therefore the higher background response in **b)**. The liquid dilution for the pulse was 10^{-2} in **a)** and 10^{-3} in **b)**. Labels on the x-axis corresponds to gas phase dilutions generated by the odor delivery system as indicated in **table S3**.

c) Three single trials from the dataset showing responses to ON and OFF stimuli with small contrast. The shaded area indicates the difference in response between test and background stimulus. The dotted line indicates the response to the control stimulus. These traces show how a small positive contrast leads to nearly perfectly invariant response (same as controls), while a small negative contrast leads to a strong decrease in calcium, much lower than the test stimulus amplitude. **d)** Control and background adapted response measure in DM2 using either a calcium or voltage sensor. Thick lines indicate the mean ΔF/F and the shaded areas sem. Liquid dilution of methyl acetate is 10^{-4} for the background and 10^{-2} for the pulse. **e)** Boxplot representing median, quartiles, min/max values and outliers of ASAP2s signals from two glomeruli. (DM2 N=19, VM7d N=23, $^*p<0.05$, Kruskal-Wallis).

f) Average PID response to methyl acetate using a liquid dilution of 10^{-2} and background airflow dilution of C6 and pulse C7 (N=4) with stimulation protocol in control (blue) and background adapted conditions (red). **g)** Same as d) for pure 2-butanone with a background airflow dilution of C7 and pulse C8 (N=4).
